# Mental health outcomes in young adolescents born preterm reveal the need for earlier screening

**DOI:** 10.1192/j.eurpsy.2026.12240

**Published:** 2026-07-17

**Authors:** Emilia Greif, Frederike Schröpfer, Jekaterina Dudko, Leonie Göbel, Rabea Kühl, Katharina Röse, Michael Lipp, Jonas Obleser, Wolfgang Göpel, Stefan Borgwardt, Léon Franzen

**Affiliations:** 1 Department of Psychiatry and Psychotherapy, University of Luebeck, Luebeck, Germany; 2 Institute of Health Sciences – Department of Occupational Therapy, University of Luebeck, Luebeck, Germany; 3 Fachklinik für Junges Leben, Diakonie Nord Nord Ost, Luebeck, Germany; 4 Department of Psychology, University of Luebeck, Luebeck, Germany; 5 Department of Paediatrics, University of Luebeck, Luebeck, Germany

**Keywords:** adolescence, DISYPS-III, long-term outcomes, low birth weight, mental health, preterm birth

## Abstract

**Background:**

Early adolescence marks a critical period for the onset of psychiatric symptoms, with 50% of mental disorders emerging before age 18. Recent evidence points to individuals being born preterm and/or with low birth weight (henceforth, PTB) having an increased risk of developing mental disorders in adolescence. Surprisingly, existing early detection and intervention strategies in adolescence are not tailored to PTB needs and profiles. The previous use of several disorder-specific instruments complicates unified, efficient screening.

**Methods:**

We analyzed data on the frequency of experiencing mental health challenges from 225 adolescents aged 10-14 years (*M* = 12.03 years) who were born very PTB (*M* = 28.03 weeks, *M* = 999.62 g) to test the applicability of the diagnostic system for mental disorders for children and adolescents – III (DISYPS-III) to PTB adolescents.

**Results:**

A confirmatory factor analysis replicated the DISYPS-III’s factorial structure and internal consistency for 10- to 14-year-old PTB adolescents. Overall, PTB adolescents reported significantly more mental health challenges than the DISYPS-III community norm sample. The instrument’s transdiagnostic scales provided a better relative fit to these challenges compared to the disorder-specific scales. Results also replicated specific findings of mental health challenges from other PTB cohorts and countries, such as a higher number of comorbidities in adolescents officially diagnosed with the inattentive presentation (ADD) of ADHD.

**Conclusions:**

These findings of an elevated psychological burden suggest a need for structured PTB screening of long-term mental health outcomes in adolescence that also includes 10-year-old PTB individuals, with the DISYPS-III being a suitable screening instrument.

## Introduction

Thanks to medical advances in recent years, the survival rate of preterm-born children has more than doubled compared to rates prior to 1990 [1]. The positive consequence is a steadily growing population of surviving preterm-born individuals. Though crucial questions around their long-term quality of life and sequelae of being born preterm and/or with low birth weight (PTB) remain.

In recent years, evidence for PTB as a risk factor for developing mental health problems in adolescence or adulthood has been mounting [2–4]. PTB individuals have been found to experience increased rates of mental health challenges and disorders [4–6]. Symptoms of attention deficit hyperactivity disorder (ADHD), autism spectrum disorder (ASD), and anxiety disorder (ANX) [6–9] have been summarized as the “preterm behavioral phenotype (PBP)” [2, 10], though mechanisms remain elusive [11]. Two meta-analyses have provided further support for more frequent anxiety and ASD diagnoses in PTB individuals [12, 13]. More research has reported elevated symptoms of other disorders, including mood [9], depressive [14], and obsessive-compulsive disorder (OCD) [15], as well as higher prevalence rates of neurodevelopmental and learning disorders [16]. Another study reported a strong association between prematurity and ADHD symptoms, alongside no identified risk factors at the family level, which the authors interpreted as evidence for a causal relationship [17]. While these separate findings altogether point to numerous elevated symptom areas, investigations of comorbidities and co-occurrence of symptoms in the same PTB individuals have been scarce [10]. As opposed to the aforementioned disorder-specific findings on mental health, it is also a current topic of contention whether a transdiagnostic approach might be more fitting in early adolescence, a time when symptoms and their severity may be transient and not always allow for unequivocal allocation to a disorder [18].

From a neurodevelopmental perspective, in utero development, birth, and adolescence constitute sensitive periods in neurological and psychological development, with generally increased vulnerability to developing mental disorders in adolescence [18–20]. Approximately 75% of mental disorder incidences peak between 12 and 25 years of age [18]. During the adolescent period, neuronal plasticity is assumed to be elevated, facilitating experience-based alterations in the brain [19]. Thus, adolescents might not only be more vulnerable to developing psychological disorders during sensitive periods but may also be more receptive to prevention and early intervention strategies [18].

Developing a better understanding of the specific profile of mental health challenges of PTB individuals during early adolescence could provide a pathway toward improved long-term outcomes and support. To move toward such improvements, we need comprehensive instruments that probe a wide range of mental health symptoms in detail and are suitable for young adolescents after PTB in accordance with the WHO’s definition of adolescence (10–19 years; [21]). Such instruments could assist with the much-needed early detection of mental health challenges [22]. However, heterogeneity in instruments administered to PTB populations, paired with some investigators solely relying on external assessments, represents a major challenge for advancing early screening and detection in this group.

As well, commonly used instruments for assessing a range of mental health outcomes within the same instrument are only designed for self-report use from age 11 onwards (e.g., DISYPS-III; Youth Self Report [YSR], [23]; Strengths and Difficulties Questionnaire [SDQ], [24]) or do not focus on symptoms clearly tied to separate clinical disorders (SDQ [24]). Therefore, these instruments could not be used for comparing transdiagnostic to disorder-specific approaches from age 10 onwards, which is key to the present investigation. Contrarily, the DISYPS-III screening instrument’s global and primary subscale structure allows for contrasting both approaches but, so far, has only been validated for use from age 11 onwards. This would make a 10-year-old too young for diagnostic self-report tools of common early detection frameworks [18, 25]. However, German students are required to transfer from primary to secondary school at age 10 [26]. This transition in the students’ social environment and increased academic demands may be accompanied by heightened stress [27, 28]. Therefore, comprehensively assessing the mental health challenges of 10-year-old PTBs appears particularly relevant.

Taken together, the present study aims to (1) characterize the mental health outcomes of PTB adolescents aged 10–14 years using the DISYPS-III screening instrument and thereby (2) extend this instrument’s construct validation to children aged 10 and PTB populations, and (3) compare the applicability of a transdiagnostic approach to a disorder-specific approach for early screening. We hypothesize that PTB adolescents will report significantly more mental health challenges than the DISYPS-III’s representative community norm sample, and we will find a symptom structure akin to a PBP, including elevated symptoms of ADHD, ASD and ANX, in a large German PTB cohort aged 10–14; assuming the instrument’s ability to capture the mental health status of the sampled 10-year-olds. The findings could increase our understanding of the co-occurring mental health challenges young PTB adolescents face and support tailoring future early detection and intervention strategies to PTB’s specific needs [4].

## Methods

### Participants

We recruited 230 adolescents from the German Neonatal Network (GNN) cohort. Inclusion criteria at birth were GA < 29 + 0 and/or a birth weight of <1500 g. The present study required participants to be aged 10–14 at the time of the follow-up assessment. Data from five adolescents were excluded due to missing DISYPS-III data. Thus, the final sample comprised 225 PTB adolescents (*N*_female_ = 96 [42.7%], *M*_age_ = 12.03, *SD*_age_ = 1.24). GA, birth weight, and birth weight percentile are in line with other GNN subsamples (e.g., [29]; *M*_GA_ = 28.03, *SD*_GA_ = 2.49 weeks, range [22 + 5; 34 + 3]; *M*_bw_ = 999.62 g, *SD*_bw_ = 296.30, range [350; 1496]; *M*_bw%_ = 41.12, *SD*_bw%_ = 28.42, range [0; 95]; for details on characteristics by sex and age see Table 1). An a posteriori sensitivity analysis in G*Power (Version 3.1, [30]; α = 0.05, 1-β = 0.95) indicated that our sample size would be sufficient to detect an effect size of f^2^ ≥ .11, corresponding to a medium effect.

**Table 1.** Demographic data Table 1. long description.

	Total*N* = 225	Sex		Age	
		Female*N* = 96 (42.7%)	Male *N* = 129 (57.4%)	Age 10 *N* = 46	Age ≥ 11*N* = 179
Age, M (SD)	12.03 (1.24)	12.11 (1.33)	11.97 (1.17)	10.48 (.32)	12.42 (1.07)
GA, M (SD)	28.03 (2.49)	27.95 (2.62)	28.09 (2.4)	28.57 (2.64)	27.89 (2.44)
Birth weight, M (SD)	999.62 (296.30)	964.24 (290.93)	1025.95 (298.64)	1079.52 (310.52)	979.09 (298.89)
Birth weight percentile, M (SD)	41.12 (28.42)	42.20 (29.19)	40.31 (27.93)	43.28 (30.88)	40.56 (27.82)
Multiple birth status, N (%)					
Single	123 (54.67)	50 (52.08)	73 (56.59)	24 (52.17)	99 (55.31)
Twins	77 (34.22)	35 (36.46)	42 (32.56)	15 (32.61)	62 (34.64)
Triplets	15 (6.67)	7 (7.29)	8 (6.2)	2 (4.35)	13 (7.26)
Dyslexia diagnosis	23 (10.2%)	10 (10.4%)	13 (10.1%)	5 (10.9%)	18 (10.1%)
ADHD diagnosis	16 (7.1%)	6 (6.3%)	10 (7.8%)	4 (8.7%)	12 (6.7%)
Inattentive presentation diagnosis (ADD)	20 (8.9%)	8 (8.3%)	12 (9.3%)	2 (4.3%)	18 (10.1%)

*Note:* Age given in years, gestational age (GA) given in weeks, birth weight given in grams. M = Mean, SD = standard deviation.

### Instruments

Mental health outcomes were assessed with the screening questionnaires of the diagnostic system for mental disorders according to ICD-10 and DSM-5 for children and adolescents – III (DISYPS-III; [31]). These standardized questionnaires comprise 50 questions to assess symptoms across a broad range of mental health disorders, including ADHD, ANX, ASD, depression (DEP), developmental disorder (DVM), disorders of social behavior (SB), and OCD. Note that scoring high on these scales does not automatically correspond to a clinical diagnosis but rather points toward a specific symptom burden. In addition to these seven disorder-specific subscales, items can be categorized more coarsely into three global subscales of externalizing (EXT), internalizing (INT), and attachment-related (CON) problems. Answers are given on a 4-point Likert-scale ranging from 0 (never) to 3 (always).

The DISYPS-III screening questionnaires are available as self-rating (SBB) and external assessment (FBB), though items remain identical with only a change of pronouns. Since the manual allows for different implementations and sources of information, including “clinical exploration”, we used self-ratings as well as external assessments (for details, see Procedure). The DISYPS-III screening has been validated in a representative German general population sample (*N* = 950; range_age_ = 11.0–17.11, *M*_age_ = 14.22, *SD*_age_ = 1.79; 46.9% male).

### Procedure

All assessments were conducted as part of the second GNN follow-up appointment, which selects a demographically balanced subsample of adolescents between 10 and 14 years of age who had already taken part in the first follow-up when aged 5–7. Testing took place across 11 different university hospitals and medical facilities in Germany in 2024–25. Test environments provided a quiet atmosphere in a separate room and a selected set of trained experimenters.

The self-report mental health screening questionnaire of the DISYPS-III was conducted in a 1-on-1 setting [31]. All Items were read out loud to participants to ensure the best possible understanding, irrespective of reading or other cognitive abilities. This step became necessary due to some items being phrased in a complicated manner, the heterogeneous nature of cognitive outcomes, and increased ratios of learning difficulties in PTB populations. In a few cases where cognitive abilities would not have allowed for an unambiguous understanding of the questions (*N* = 10), for instance, when asking children with lower intelligence quotient (IQ), cerebral palsy, or trisomy, we asked their guardians for an external assessment. To depict the most realistic mental health status possible, researchers factored in external observations from the testing situation by carefully adjusting selected self-ratings that clearly contradicted the observed behavior or accompanying verbal reports according to the clinician’s assessment (e.g., a child reporting no fidgeting at any time, while visibly fidgeting frequently during testing and interview; for details on adjustment criteria, see Supplementary Material S1). Overall, we adjusted 99 of 10.800 responses (0.92%). These adjustments affected 53 adolescents, with the items S01 (6x), S02 (7x), S03 (7x), S30 (8x), and S34 (6x) being adjusted several times. These items correspond to the ADHD subscale (S01-S03) or address eating disorders (S30) and learning difficulties (S34). Hence, the implementation in our study differed from the standardized procedure in the following points: (1) we also included 46 children at age 10–10.11, as opposed to the norm sample starting at 11.0; (2) we excluded item 28 (suicidal ideation), as the non-psychiatric context of our study could not provide the required ad hoc psychological support, which the question might have called for; (3) items were read out loud to the adolescents; and (4) additional external assessments and clinical observations were factored into the final scores. Separately, guardians were asked about existing diagnoses (e.g., psychiatric, learning disorders, etc.).

The overall procedure during this follow-up appointment included assessments of somatic, cognitive (e.g., reading and visuo-motor integration), and mental health parameters. It lasted approximately 120 minutes, including 15 minutes for the mental health questionnaire. Ethical approval was obtained from the ethics committee of the University of Luebeck (# 2023-812_2).

### Statistical analyses

All statistical analyses were performed in RStudio (Version 2025.05.1 + 513; [32] and MATLAB (Version 2025a; [33]). Birth weight percentiles were calculated using the Fenton 2025 Growth Calculator for Preterm Infants on PediTools ([34] accessed via https://peditools.org/fenton2025/).

To evaluate the general mental health status in PTB adolescents, we assessed ratings on each DISYPS-III global and primary subscale. These ratings served as the basis for calculating an arithmetic mean (quotient), considering the removal of item 28, which affected the total, DEP, and INT scales. The quotient can be interpreted as the average frequency of occurrence for a group of symptoms reported by an adolescent on the corresponding scale (range 0–3). This indicator benefits from not relying on potentially biased norm distributions as a reference point for assessing clinical relevance and from no difference between the item phrasings of self-rating (SBB) and external assessment (FBB) questionnaires. Subsequently, we separated the quotients into two sets: One set including only PTB adolescents with a minimum age of 11.0 (henceforth, 11+; *N =* 179), which parallels the representative community sample of the DISYPS-III self-rating questionnaire [31], and a second sample including all data. The 11+ subsample served as a baseline for comparisons of the questionnaires’ properties after inclusion of the 10-year-olds.

To evaluate the DISYPS-III’s internal consistency and reliability in a PTB cohort, we calculated Cronbach’s Alpha (package psych::alpha; [35]) within the 11+ subsample and compared these results to those obtained from the full dataset including all adolescents aged 10–14. Additionally, to examine the specific mental health challenges reported by 10-year-old PTB adolescents and to aim for robust scores despite the smaller subsample size (*N* = 46), we bootstrapped (package base R::replicate; [32]) the younger group’s mean DISYPS-III total scores 1000 times with replacement.

**Figure 1.**
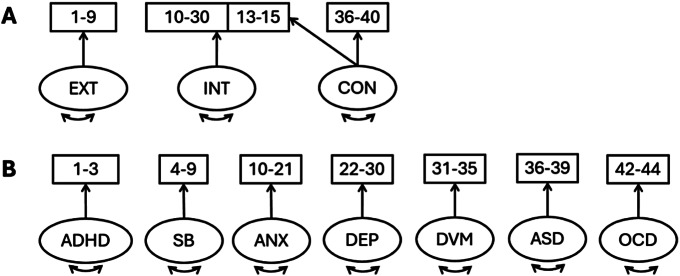
Measurement models for a 3-(A) and 7-(B) factorial solution representing the DISYPS-III global and primary subscales. *Note*: Rectangles represent manifest variables, with numbers indicating questionnaire items. Oval forms represent latent factors. EXT, externalizing problems; INT, internalizing problems; CON, attachment-related problems; ADHD, attention deficit and hyperactivity disorder; SB, disorders of social behavior; ANX, anxiety disorder; DEP, depression; DVM, developmental disorder; ASD, autism spectrum disorder; OCD, obsessive-compulsive disorder. Figure 1. long description.

To replicate the factorial structure of the DISYPS-III norm sample and assess the structural validity in the present PTB sample’s ratings, we used several confirmatory factor analyses (CFAs) on data from the entire sample and the 11+ subsample, respectively. Factor loadings of each item in a three-factor solution representing the global subscales (Figure 1A) and a seven-factor solution representing the primary subscales (Figure 1B) were extracted from the instrument’s manual [31]. These solutions were obtained by the instrument’s original authors from the community norm sample using exploratory factor analysis (EFA) and principal component analysis with Varimax rotation and formed the basis for the presented CFAs. We calculated each of the three- and seven-factor solutions for the full and 11+ dataset separately (lavaan::cfa; [36]), using full information maximum likelihood (FIML) to impute missing values. The following model fit indices and information criteria were used to compare the different models’ ability to reflect our PTB data. The Comparative Fit Index (CFI; [37]) and the Tucker–Lewis index (TLI; [38]) compare the specified model fit to the null model (all covariances set to zero), with higher scores indicating better model fit. Root Mean Squared Error of Approximation (RMSEA; [39]) and Standardized Root Mean Square Residual (SRMR; [40]) assess differences between model-predicted and actual values, with lower scores indicating better model fit. The cut-offs for acceptable RMSEA and SRMR fit were set to ≤ .06 and < .08, respectively [41, 42]. While RMSEA and SRMR values met these thresholds, the CFI and TLI values fell below the conventional cut-off of ≥ .90, indicating only moderate model fit. As well, we used Akaike’s Information Criterion [43] and the Bayesian Information Criterion [44] to assess model fit. Although numerical values are arbitrary, better model fit is represented by lower scores.

To provide a differentiated picture of the mental health status of the present PTB sample in adolescence, we calculated Bonferroni-corrected independent t-tests comparing the mean quotients of all global and seven primary subscales between the PTB and DISYPS-III norm samples [45]. Characteristics available for the instrument’s community norm sample were sample size, mean and standard deviation (SD) for the global and each subscale. As a complement to these tests, we extrapolated the available information and knowledge on general right-skewness on similar rating scales by simulating hypothesized underlying distributions of single-participant data using gamma functions that matched the available mean and SD information from the community norm sample most closely. Since the present distributions of our PTB mental health ratings also showed right-skew, we compared groups using three non-parametric tests (Wilcoxon rank-sum test [wilcox.test] and permutation analyses of mean or median differences with 10,000 resamples, respectively [coin:oneway_test and coin:median_test]).

The instrument’s manual also reports sex differences for the norm sample. Therefore, we quantified the evidence for sex differences within each subscale by calculating complementary Welch’s two-sample t-tests (two-sided) and Bayes Factors (BayesFactor::ttestBF; [46]). Q-Q plots indicated normality of these distributions stemming from the collected PTB sample.

To confirm the specific role of hyperactivity within the ADHD symptom cluster, we grouped participants by self- and/or guardian-reported official diagnosis of ADHD or its inattentive presentation (ADD) and compared these two subtypes’ primary subscale quotients.

Lastly, to explore the possibility of a PBP, we conducted an EFA (lavaan::efa; [36]) on the same sample used for all other analyses, meaning that this step must be considered exploratory and interpreted with appropriate caution. The number of factors was specified to k = 3, based on a scree plot (Supplementary Figure S2), and to k = 6, based on parallel analysis. We systematically evaluated both solutions as these not only stem from statistical results but also fit our theoretical framework of contrasting transdiagnostic and disorder-specific approaches. Congruently, the DISYPS-III also uses two-factor structures, representing transdiagnostic and disorder-specific approaches. In line with the DISYPS-III manual, factor loadings of a > .25 were considered for interpretation. An overview of sample sizes included in all analyses is presented in the Supplementary Materials (Supplementary Table S3).

## Results

The present study extended the validation of the DISYPS-III screening questionnaires for a quick screening of mental health in young adolescents aged 10–14 after PTB and characterized their mental health challenges. Specifically, our results show good overall internal consistency without a reduction in this consistency when including 10-year-olds, and significantly elevated levels of mental health symptoms in PTB adolescents.

### Structural analyses confirmed DISYPS-III internal consistency

The fit indices of the DISYPS-III’s seven- and three-factorial CFA models conducted with data from all participants suggest similar structural validity when including ratings of 10-year-old participants (Table 2; for details, see Supplementary Tables S4–S6). Both CFAs conducted across the entire age span (10–14) displayed accurate fit according to RMSEA and SRMR error indicators and moderate structural fit according to CFI and TLI indices. The models that included the entire age span showed better fit when compared to those including data from 11+ year olds (Table 2). Information criteria and Likelihood Ratio Tests demonstrated the superiority of the three-factor solution that focuses on the three global subscales EXT, INT, and CON (*ξ*^*2*^*Δ* = 293.34, *p* = .007). Two complementary sensitivity analyses comparing (1) the effect of clinician-adjusted ratings across self- and external ratings and (2) adjustments on self-ratings only (i.e., excluding entirely externally rated individuals; *N* = 215) resulted in negligible differences in fit indices, underscoring robustness against adjustments of self-ratings by clinicians and the combination of self- and external caregiver ratings (Supplementary Tables S7a–c).

**Table 2.** Fit indices for CFAs Table 2. long description.

CFA	Sample	CFI	TLI	RMSEA	SRMR	AIC	BIC
3 factors	10–14	.746	.727	**.060**	**.072**	14515	14884
7 factors	10–14	.766	.747	**.053**	**.070**	17904	18396
3 factors	11+	.717	.698	.064	**.078**		
7 factors	11+	.720	.697	**.060**	**.077**		

*Note:* N = 225 for the entire dataset of adolescents aged 10–14; N = 179 for adolescents aged 11+. Missing values were imputed using full-information maximum likelihood. CFI = Comparative Fit Index; TLI = Tucker–Lewis Index; RMSEA = Root Mean Square Error of Approximation; SRMR = Standardized Root Mean Square Residual; AIC = Akaike’s Information Criterion; BIC = Bayesian Information Criterion. Bold values indicate that the cut-off for acceptable fit was met. For detailed factor loadings and covariances between factors, see Supplementary Tables S4–S6.

In line, the internal consistency of most subscales remained within a similar range compared to the DISYPS-III norm sample and did not improve substantially across all DISYPS-III subscales in the 11+ dataset (Table 3). We observed deviations only for the subscales OCD, ASD, SB, and CON, with OCD being more consistent (*Δα* = .12), while the other subscales showed less consistency in our specific population (*Δα* = .06–.21; Table 3).

**Table 3.** Descriptive statistics of quotients and internal consistency for each DISYPS-III subscale Table 3. long description.

Scale	M_all_ (norm sample)	SD_all_ (norm sample)	95% CI lower	95% CI upper	Internal consistency (α_all_)	α_11+_ (norm sample)	Δ α (α_11+_ vs. α_all_)	Δ M (M_all_ vs. M_norm_)
Total	.47 (.33)	.29 (.26)	.43	.51	.90	.89 (.91)	−.01	.14
EXT	.57 (.36)	.41 (.34)	.51	.62	.72	.73 (.76)	.01	.21
INT	.51 (.38)	.36 (.34)	.46	.56	.85	.85 (.87)	0	.13
CON	.34 (.23)	.34 (.34)	.30	.39	.63	.61 (.79)	−.02	.11
ADHD	1.01 (.59)	.77 (.56)	.91	1.11	.70	.71 (.70)	.01	.42
ANX	.56 (.40)	.39 (.35)	.51	.61	.77	.75 (.78)	−.02	.16
ASD	.40 (.23)	.47 (.40)	.34	.46	.54	.59 (.75)	.05	.17
DEP	.43 (.37)	.42 (.41)	.38	.49	.76	.76 (.81)	0	.06
DVM	.51 (.20)	.47 (.30)	.45	.57	.51	.51 (.50)	0	.31
OCD	.40 (.26)	.54 (.40)	.33	.48	.61	.65 (.49)	.04	.14
SB	.35 (.24)	.33 (.30)	.30	.39	.59	.63 (.67)	.04	.11

*Note:* Internal consistency is given as Cronbach’s Alpha [47], missing item values were handled using pairwise deletion [35]. The 11+ dataset included only adolescents aged a minimum of 11.0 years (N = 179). Characteristics of the official norm sample (N = 950; [31]) are depicted in brackets. M, Mean; SD, standard deviation; CI, confidence interval; EXT, externalizing problems; INT, internalizing problems; CON, attachment-related problems; ADHD, attention deficit and hyperactivity disorder; ANX, anxiety disorder; ASD, autism spectrum disorder; DEP, depression; DVM, developmental disorder; OCD, obsessive-compulsive disorder; SB, disorders of social behavior.

### PTB adolescents reported more mental health challenges

**Figure 2.**
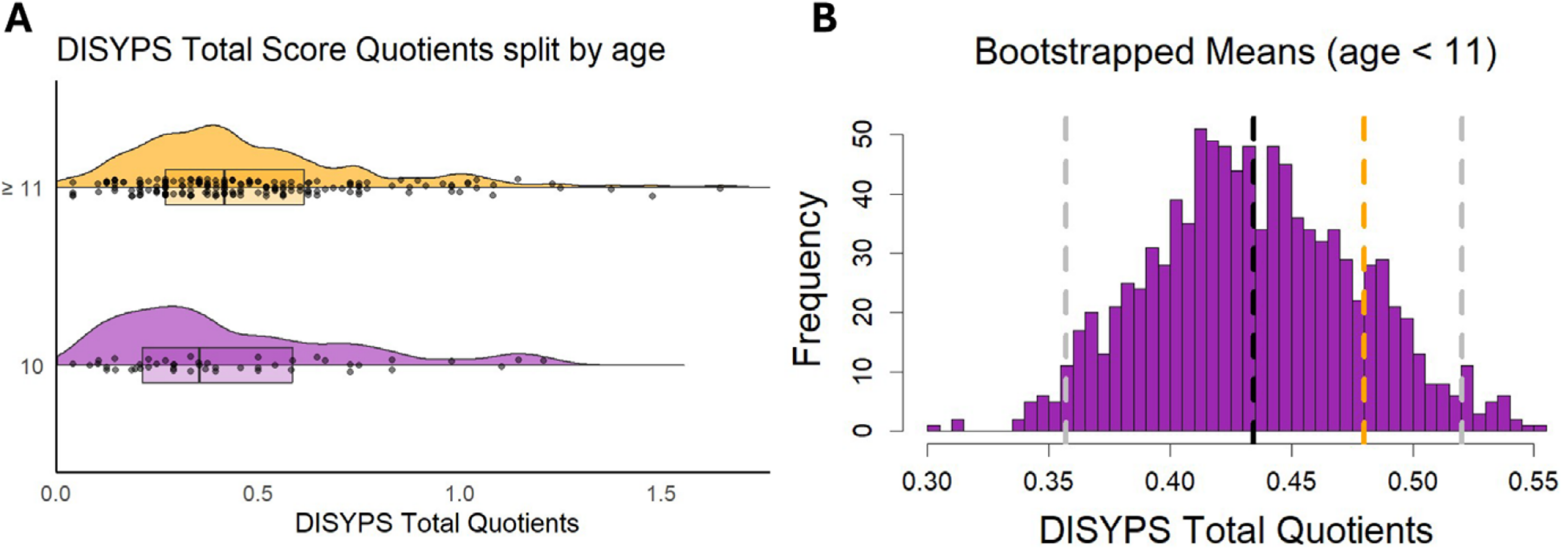
DISYPS-III total quotients split by age of the PTB adolescents at the time of testing. *Note*: (A) Raincloud plot of total scale quotients comparing adolescents aged 10 (*N* = 46, purple) to those aged 11+ (*N* = 179, yellow). Boxplots indicate median and interquartile range. (B) Histogram of 1000 bootstrapped total scale quotient means of the original sub-dataset of 10-year-olds (*N* = 46). Dashed lines indicate the grand mean (black, *μ* = .44) and 95% confidence interval (gray, 95% CI [.36, .52]) for the 10-year-olds, while the dashed orange line depicts the grand mean of the 11+ year olds. Figure 2. long description.

Mental health challenges reported by the 10-year-old PTB individuals matched those of older PTB adolescents, as our data provide evidence of similar mean quotients on any subscale in both age groups (Table 4; Figure 2A,B). This result is corroborated by results from a robust bootstrapping analysis showing that the grand means did not differ significantly, as the mean reported by 11+ PTBs falls clearly within the confidence interval of the 10-year-olds’ group mean (Figure 2B). Therefore, in the following, we present PTB adolescents aged 10–14 as one group.

**Table 4.** DISYPS-III mean quotients and their standard deviation for all subscales, split by age of the PTB adolescents at the time of testing Table 4. long description.

	Total	Global subscales			Primary subscales						
		EXT	INT	CON	ADHD	ANX	ASD	DEP	DVM	OCD	SB
Age 10 M (SD)	.44 (.30)	.54 (.39)	.48 (.35)	.32 (.35)	1.00 (.77)	.57 (.40)	.39 (.46)	.35 (.36)	.45 (.54)	.38 (.40)	.32 (.31)
Age 11+ M (SD)	.48 (.29)	.57 (.41)	.52 (.37)	.35 (.34)	1.01 (.77)	.56 (.39)	.41 (.47)	.46 (.44)	.52 (.45)	.41 (.57)	.35 (.33)
Δ M	.04	.03	.04	.03	.01	−.01	.02	.09	.07	.03	.03
*p*-value	.365	.593	.529	.638	.942	.868	.775	.09	.404	.664	.472
Bayes Factor	.262	.201	.211	.198	.178	.180	.184	.531	.264	.200	.222

*Note:* Statistical significance was tested using Welch’s two-sample t-test as well as Bayes Factors. A Bayes Factor between 0 and 1 is interpreted as evidence favoring H_0_, 1–3 as ambivalent, and > 3 as evidence favoring H_1_ [48]. M, Mean; SD, standard deviation; EXT, externalizing problems; INT, internalizing problems; CON, attachment-related problems; ADHD, attention deficit and hyperactivity disorder; ANX, anxiety disorder; ASD, autism spectrum disorder; DEP, depression; DVM, developmental disorder; OCD, obsessive-compulsive and tic disorders; SB, disorders of social behavior.

**Figure 3.**
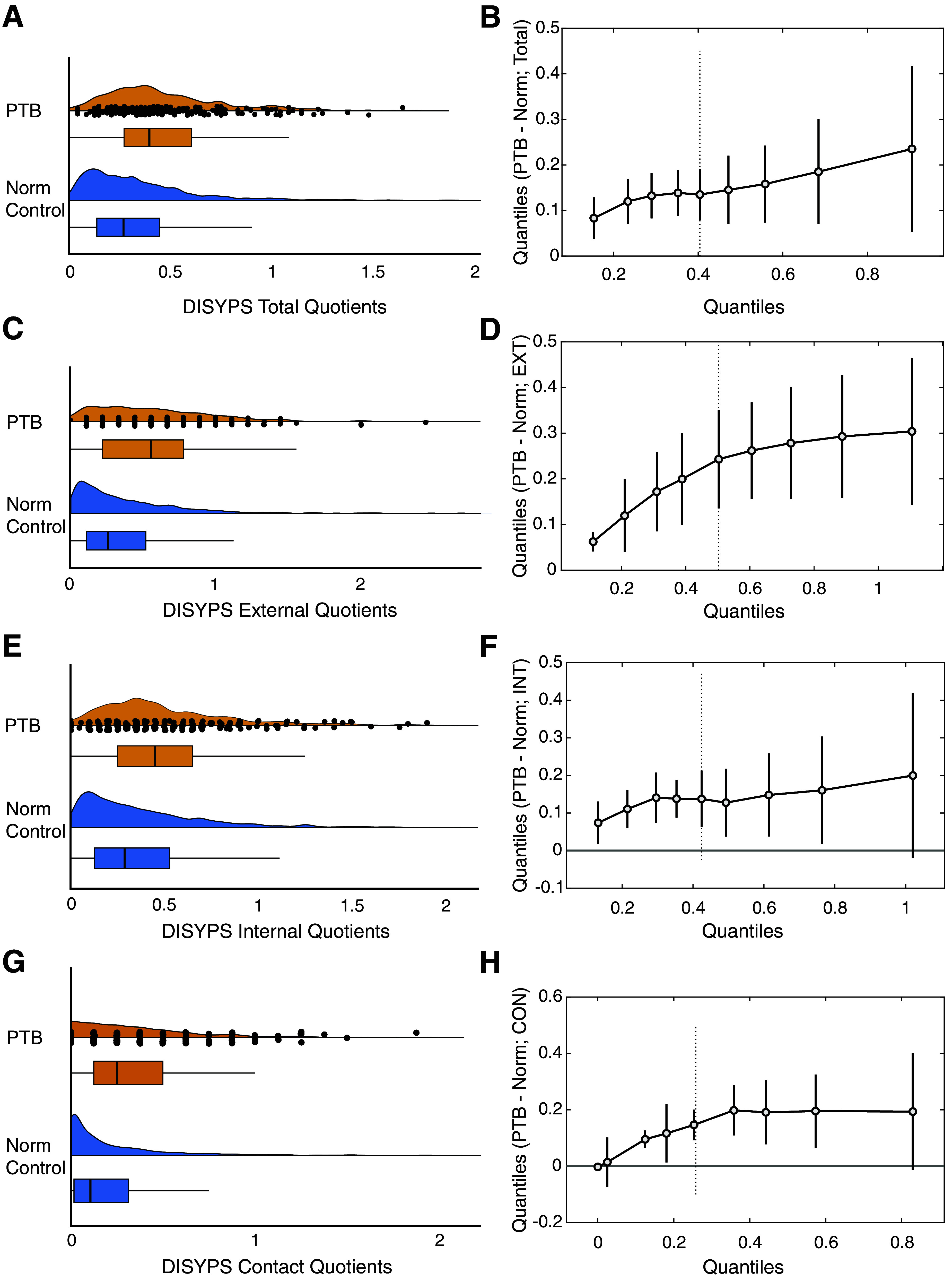
PTB adolescents reported greater mental health challenges and more extreme values compared to the representative non-clinical community sample. (A, C, E, G) *Note*: Raincloud plots of total and global subscale DISYPS-III quotients comparing the PTB (orange) to a simulated norm sample (blue) based on the statistics reported in the manual (for details, see Table 3). Global subscales include externalizing symptoms (EXT; panel C), internalizing symptoms (INT; panel E), and attachment-related behavior (CON; panel G). Boxplots display interquartile range and median. Total score quotients are equivalent to a person’s mean across all items. (B, D, F, H) Robust shift functions [49] comparing the distribution of DISYPS-III quotients between PTB adolescents and the simulated norm sample per decile. Gray dots represent the mean difference (PTB–norm) and vertical black bars depict the decile’s bootstrapped 95% confidence interval. A vertical black bar not including 0 indicates a significant difference. Figure 3. long description.

PTB adolescents reported generally elevated mental health challenges compared to the community norm sample (*ΔM* = .14, *ΔM_Wilcoxon_* = .13, 95% CI [−.17, −.10]; Tables 3, 5; Figure 3A,B). While the majority of PTB adolescents’ average responses per scale ranged from 0 to 1 (never to sometimes), a considerable number scored above 1 (often) on total scale quotients (Figure 3A). This difference is a consequence of significantly more PTB adolescents reporting higher scores compared to the simulated norm sample, which might be indicative of present mental health challenges (Figure 3B). This pattern of results also shows in the distributions of quotients on the three global scales (EXT, INT, and CON), which were revealed by the CFA as the scales with the best relative fit (Table 2; Figure 3C–H). Here, we observed a significant increase in the frequency of high scores across all global scales (Figure 3C–F), except for the highest decile of attachment-related behavior (CON; Figure 3G,H). Importantly, traditional parametric and assumption-free non-parametric approaches uniformly underlined significantly elevated symptoms across these global scales (Table 5; Figure 3).

**Table 5.** Statistical differences between the PTB sample and the norm sample on DISYPS-III scales Table 5. long description.

	Total	EXT	INT	CON	ADHD	ANX	ASD	DEP	DVM	SB	OCD
PTB M (SD)	.47 (.29)	.57 (.41)	.51 (.36)	.34 (.34)	1.01 (.77)	.56 (.39)	.40 (.47)	.43 (.42)	.51 (.47)	.35 (.33)	.40 (.54)
PTB N	225	225	225	225	225	225	225	225	224	225	214
Norm M (SD)	.33 (.26)	.36 (.34)	.38 (.34)	.23 (.34)	.59 (.56)	.40 (.35)	.23 (.40)	.37 (.41)	.20 (.30)	.24 (.30)	.26 (.40)
Norm N	950	950	950	950	950	950	950	950	950	950	950
Welch t-test T (df), *p*-value	6.64 (315) <.0001*	7.13 (301) <.0001*	4.92 (325) <.0001*	4.36 (338) <.0001*	7.71 (283) <.0001*	5.64 (315) <.0001*	5.01 (305) <.0001*	1.94 (333) .054	9.43 (267) <.0001*	4.57 (317) <.0001*	3.58 (268) <.001*
Welch t-test Δ 95% CI	[.10, .18]	[.15, .27]	[.08, .18]	[.06, .16]	[.31, .53]	[.10, .22]	[.10, .24]	[−.001, .12]	[.25, .38]	[.06, .16]	[.06, .22]
Wilcoxon W, *p*-value	71891 <.0001	70859 <.0001	78963 <.0001	86895 <.0001	68956 <.0001	76988 <.0001	98543 .069	96576 .024	63070 <.0001	89538 <.001	112502 .015
Wilcoxon Δ Estimated 95% CI	−.13 [−.17, −.10]	−.19 [−.24, −.14]	−.13 [−.17, −.09]	−.11 [−.12, −.07]	−.33 [−.44; −.26]	−.15 [−.20; −.11]	−.11 [−.2; −3.7e^5^]	−.06 [−.10; −.01]	−.24 [−.31; −.20]	−.10 [−.14; −.05]	.003 [.00; .01]
Permutation M Z, p-value	7.01 <.0001	7.51 <.0001	5.11 <.0001	4.36 <.0001	9.02 <.0001	5.86 <.0001	5.41 <.0001	2.09 .0351	11.63 <.0001	4.54 <.0001	4.31 <.0001
Permutation Med. Z, p-value	6.02 <.0001	7.50 <.0001	5.57 <.0001	3.98 <.0001	6.76 <.0001	4.83 <.0001	4.54 <.0001	2.06 .0494	10.69 <.0001	2.53 .0137	0.76 .5004

*Note:* M, Mean; SD, standard deviation; CI, confidence interval; EXT, externalizing problems; INT, internalizing problems; CON, attachment-related problems; ADHD, attention deficit and hyperactivity disorder; ANX, anxiety disorder; ASD, autism spectrum disorder; DEP, depression; DVM, developmental disorder; OCD, obsessive-compulsive and tic disorders; SB, disorders of social behavior. N was reduced for the testing of DVM and OCD subscales due to 1 and 11 missing values, respectively. Analyses compared groups using complementary parametric and non-parametric approaches. Welch t-test: Parametric Welch’s two-sample t-test. Significance is Bonferroni corrected for 11 tests, so that * indicates *p* = .0045. Only mean (M) and standard deviation (SD) from observed PTB data or the norm sample from the DISYPS-III manual served as a basis for these tests. Wilcoxon: non-parametric Wilcoxon Rank Sum Test and the estimated difference (control – PTB) and its 95% confidence interval (CI). Permutation M: non-parametric, assumption-free, approximative two-sample Fisher-Pitman Permutation Test testing for a difference between group means. Permutation Med.: non-parametric, assumption-free, approximative two-sample Brown-Mood Median Test testing for a difference between group medians. Both non-parametric tests are based on single-participant data by comparing observed PTB data (N = 225) to simulated norm participants (N = 950) that were sampled from a right-skewed gamma distribution.

**Figure 4.**
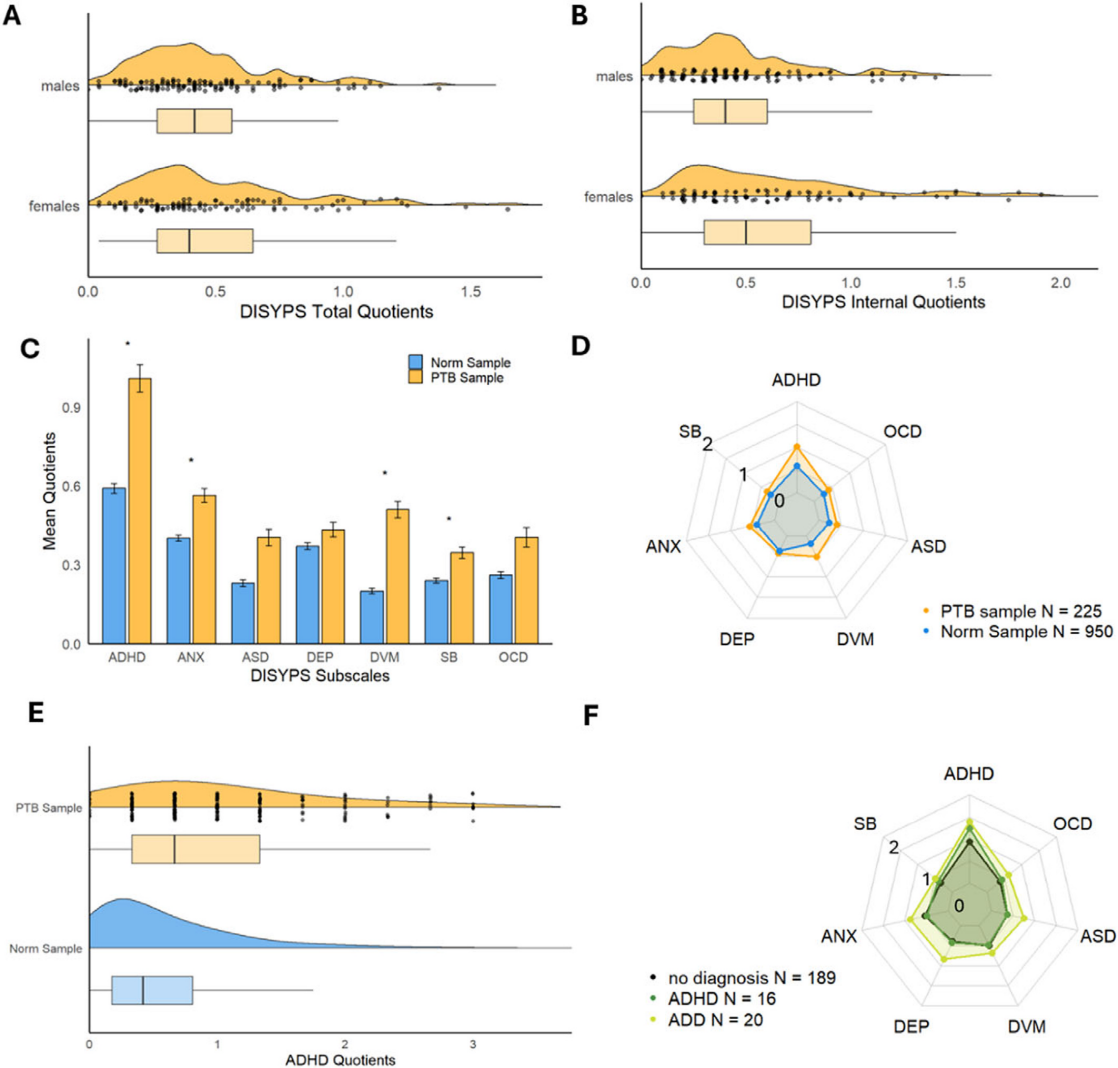
Mental health challenges split by sex and primary subscales. *Note*: (A, B) Raincloud plot of DISYPS-III total (A) and internalizing problems (B) quotients of the PTB sample split by sex. Boxplots display interquartile range and median. (C) DISYPS-III group mean quotients of all primary subscales comparing the PTB sample (orange) to the simulated norm sample (blue). Error bars in panel C indicate the standard error of the mean. For exact values see Tables 3 and 5. Significance was tested using two-sample Welch t-tests, non-parametric Wilcoxon rank-sum and permutation tests. * Indicates significant differences considering all three tests in unison. (D) Radar plot of DISYPS-III group mean quotients of all primary subscales comparing the PTB sample (orange) to the simulated norm sample (blue). (E) Raincloud plot of DISYPS-III quotients on the ADHD subscale comparing the PTB (orange) to the simulated norm sample (blue). Boxplots indicate median and interquartile range. (F) Radar plot displaying mean quotients for all DISYPS-III primary subscales split by official diagnosis of ADHD or its inattentive presentation (ADD; PTB adolescents only). Figure 4. long description.

Since sex differences are present in the norm sample and conceivable in PTB populations, we compared quotients between sexes. We found no significant difference between male and female PTB adolescents in their overall reported mental health challenges (*p* = .187, BF = .353; Table 6; Figure 4A). Female PTB adolescents, however, scored significantly higher on the INT subscale (*p* = .001, BF_INT_ = 36.37), with a subgroup of females scoring above 1 (Figure 4B). This result for internalizing symptoms is corroborated by very strong evidence in the present PTB sample (BF_INT_ = 36.37) and replicates the sex effect reported for the norm sample. Accordingly, we also found evidence for elevated levels of the two related primary subscales, anxiety and depression, in females (BF_ANX_ = 9.56, BF_DEP_ = 31.09; Table 6).

**Table 6.** Mean and standard deviation for each DISYPS-III scale split by sex Table 6. long description.

	Total	Global subscales			Primary subscales						
		EXT	INT	CON	ADHD	ANX	ASD	DEP	DVM	OCD	SB
Male M (SD)	.45 (.26)	.59 (.40)	.44 (.30)	.35 (.32)	1.07 (.75)	.50 (.35)	.43 (.49)	.35 (.31)	.56 (.51)	.34 (.52)	.35 (.32)
Female M (SD)	.50 (.33)	.53 (.41)	.61 (.42)	.34 (.36)	.92 (.79)	.65 (.43)	.37 (.44)	.54 (.52)	.45 (.41)	.48 (.56)	.34 (.33)
Δ M	.05	−.06	.17	−.01	−.15	.15	−.06	.19	−.11	.14	−.01
*p*-value	.187	.283	**.001**	.814	.145	**.004**	.384	.**002**	.082	.065	.793
Bayes Factor	.353	.255	36.37	.151	.407	9.56	.208	31.09	.564	.787	.152

*Note:* Statistical significance was tested using Welch’s two-sample t-test using a Bonferroni-corrected threshold of α < .0045. A Bayes Factor between 0 and 1 is interpreted as evidence favoring H_0_, 1–3 as ambivalent, and > 3 as evidence favoring H_1_ [48]. M, Mean; SD, standard deviation; EXT, externalizing problems; INT, internalizing problems; CON, attachment-related problems; ADHD, attention deficit and hyperactivity disorder; ANX, anxiety disorder; ASD, autism spectrum disorder; DEP, depression; DVM, developmental disorders; OCD, obsessive-compulsive and tic disorders; SB, disorders of social behavior.

### ADHD symptoms were most prominent

A more differentiated picture of symptom frequency can be drawn by examining the seven primary subscales. Although the seven-factor solution of primary subscales showed a lower fit to our data, its model fit indices were only slightly lower than those for the three-factor solution (Table 2). The primary subscales revealed significantly elevated scores of the PTB group compared to the representative non-clinical norm sample on all but the ASD, DEP, and OCD primary subscales, which underlines the general presence of mental health challenges in our PTB population (Tables 3, 5; Figure 4C,D; Supplementary Figures S8–S13).

Particularly, symptoms of ADHD were most prominent in the sampled PTB adolescents (*M*_PTB_ = 1.01*, M*_norm_ = .59, *ΔM* = .42; Figure 4C, D, Supplementary Figures S8–S13). Similar to the identified differences in global subscales (Figure 3), greater dispersion in the PTB sample stems from PTB adolescents more frequently reporting scores on the higher end of the subscale, including the maximum value (3 = “always”; Figure 4E). PTB adolescents officially diagnosed with ADHD (7.1%) or its inattentive presentation (ADD; 8.9%, see Table 1) also scored higher on the primary ADHD subscale than those diagnosed with neither (Figure 4F), which confirms that the DISYPS-III captures symptoms of this disorder in our PTB group. Interestingly, those diagnosed with the inattentive presentation type (ADD), compared to their peers with an ADHD diagnosis including hyperactivity, reported significantly higher total mental health challenges (*W_Wilcoxon_ =* 239.5*, p* = .01) and a higher level of comorbidity with other symptoms across all other primary subscales (Figure 4F). This suggests that the DISYPS-III may allow for capturing differences between symptoms of slightly varying diagnostic categories.

### DISYPS-III captured aspects of neurodevelopmental disorders

**Figure 5.**
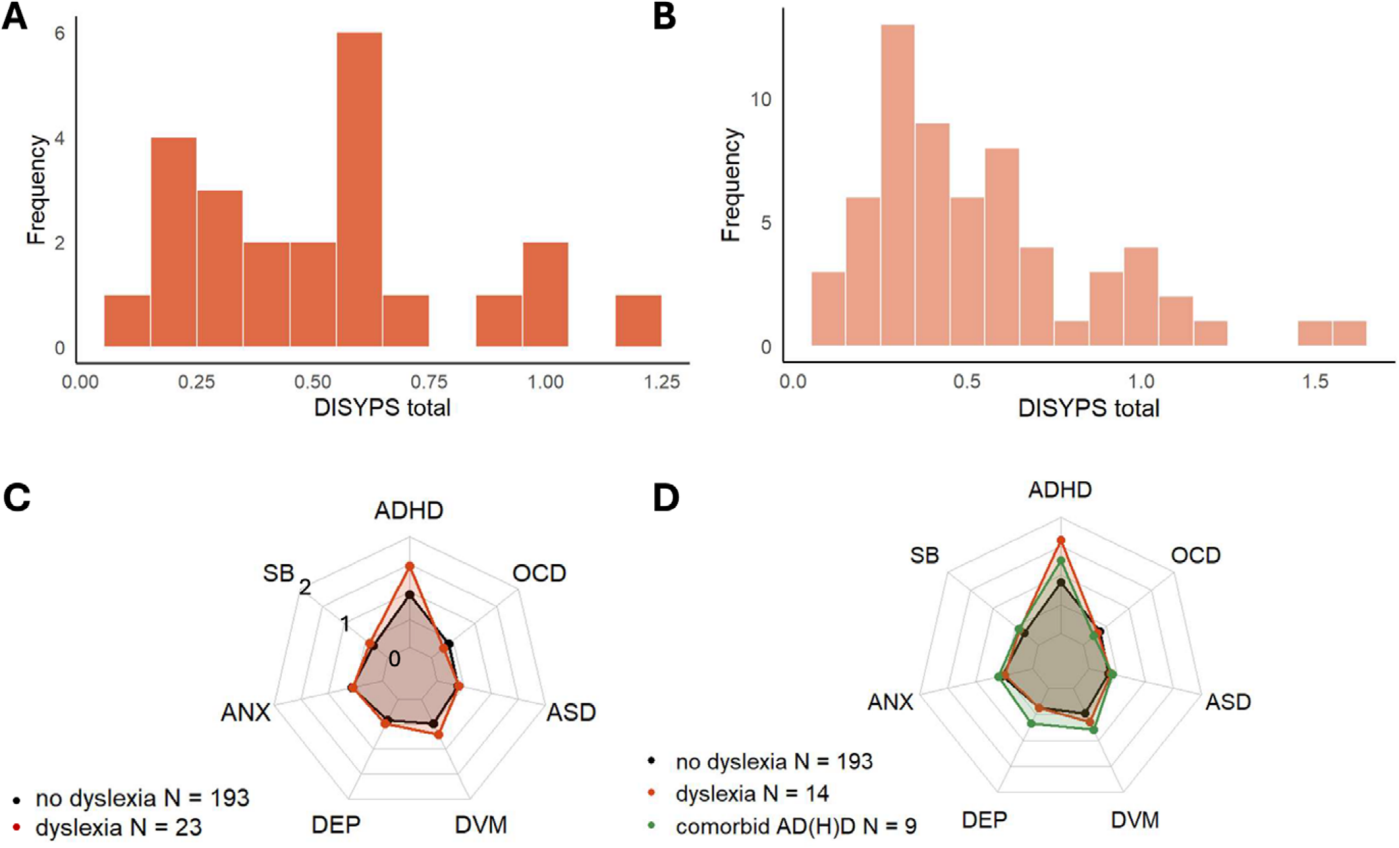
Mental health challenges vary with neurodevelopmental disorders. *Note*: (A) Histogram depicting the frequency of DISYPS-III total quotients for the PTB group with an official dyslexia diagnosis (*N* = 23) (B) and for the entire group of those diagnosed or suspected of having dyslexia (*N* = 62). (C) Radar plot comparing primary subscale mean quotients between PTB adolescents with (red) and without (black) a dyslexia diagnosis. (D) Radar plot comparing primary subscale mean quotients between PTB adolescents with a diagnosis of dyslexia only (red), dyslexia and AD(H)D (green), and no dyslexia diagnosis (black). Information on dyslexia diagnosis was missing for several datasets (*N* = 9), excluded from these graphs. Figure 5. long description.

We also found the quotient for DVMs to be among the most elevated primary symptom clusters, in line with the literature (e.g., [16]). Here, we used reading difficulties (i.e., dyslexia) as an example to examine the DISYPS-III’s ability to capture differences in mental health challenges in relation to neurodevelopmental disorders. From the entire sample, 23 (10.2%) adolescents had already received a dyslexia diagnosis, and another 39 (17.3%) self-reported ongoing assessments for dyslexia or substantial difficulties in reading and writing that go far beyond those of their peers. Information on dyslexia diagnosis was missing for nine datasets, excluded from the following analyses. Total mental health challenges were substantially elevated in the group of PTB adolescents with diagnosed dyslexia and those with suspected reading difficulties when compared to the norm sample (*M*_norm_ = 0.32, *SD* = 0.26; *M*_diagnosis_ = 0.53, *SD* = 0.297; *M*_suspected_ = 0.55, *SD* = 0.353). Though their total challenges were only slightly higher compared to PTB adolescents without reading difficulties (*M*_without_ = 0.47, *SD* = 0.33; Figure 5A,B). Nine of the adolescents diagnosed with dyslexia had also received a comorbid diagnosis of AD(H)D, in line with documented substantial comorbidities of up to 48% between both diagnoses ([50, 51]; Figure 5D). Higher DISYPS-III ADHD and DVM quotients in the diagnosed dyslexia group confirmed this comorbidity in the present PTB sample (Figure 5D).

### Evidence for a partial PBP, including anxiety and ASD

Elevated symptoms of ADHD, anxiety, and ASD in preterm groups have been postulated to form the PBP. In the present EFA, we did not find evidence supporting a fully conjoint PBP factor structure comprising all three domains (for details, see Supplementary Table S14). The first and second factors of the EFA simply differentiated internalizing and externalizing symptoms, while the third factor summarized items measuring ANX, DVM, and ASD. The latter is partially in line with the global CON scale (Supplementary Table S14). However, the current EFA grouped more anxiety-related and ASD items within the same factor, indicating that PTB adolescents who reported high scores on the ASD items also tended to report high scores on the ANX items, but not necessarily on the ADHD-related items. The latter items were fully represented in the first factor, which captured externalizing symptoms. Hence, our data only support a partial PBP of ANX and ASD without ADHD symptoms. Given the exploratory nature of our study, these data should be viewed as preliminary and suggest only a partial manifestation of the PBP, specifically one combining ANX and ASD symptoms, but excluding ADHD-related features. This pattern warrants further investigation in independent samples to determine whether it represents a stable feature of the PBP across PTB samples.

## Discussion

The present study aimed to (1) characterize the mental health outcomes of a large sample of young adolescents aged 10–14 from the longitudinal GNN cohort (*N* = 225) and (2) extend the DISYPS-III screening instrument’s construct validation to children aged 10 and PTB populations. Our results demonstrate generally elevated mental health challenges in young adolescents after PTB compared to the simulated community norm sample. This result did not differ between 10-year-old and 11–14-year-old adolescents. It was brought forth by a qualified replication of structural aspects of the DISYPS-III self-rating screening questionnaire and underlines the importance of early mental health screening in PTB populations.

Recent discussions of earlier transdiagnostic screening of mental health [18] have raised awareness of the need for tools that can support this goal already during early adolescence. The DISYPS-III is being validated in a large community and a small psychiatric sample with individuals aged 11 and older, suggesting its suitability for screening purposes in these groups. The present results of a similar factorial structure and internal consistency in 11+-year-olds after PTB, compared to the manual’s community norm sample, extend the existing factorial validation and reliability to the PTB population. Crucially, our results extend the structural validation from 11+ to 10-year-old PTB adolescents based on improved fit indices (CFI, TLI, RMSEA, and SRMR) alongside similar internal consistency.

While these improvements in fit, when including the youngest PTB adolescents, signal a comparable structure within the PTB group and the relevance of a joint evaluation of all PTB adolescents aged 10–14, this extension of the DISYPS-III construct validation is not unequivocal. Overall CFA results show qualified support of the DISYPS-III factor structure, as developed in a general community sample, with its fit indices indicating a moderate and error indices an accurate fit. Together, these results suggest that while the broad architecture of mental health symptoms in PTB adolescents aligns with that of the general population, the specific manifestation of these symptoms deviates due to this group’s unique developmental context, reflected in the moderate model fit. Important in the present context is that the fit was not affected by solely focusing on self-rated responses, which underlines a somewhat altered manifestation in PTB adolescents. Consequently, our findings imply that the DISYPS-III self-rating screening tool is applicable to PTB populations and the examined young age range, while its precision could benefit from future refinements tailored to PTB groups.

Including 10-year-olds might be particularly beneficial, since the first years of adolescence constitute a critical developmental phase characterized by typical stressors of early adolescence, such as increased academic demands and peer rejection [52]. The youngest adolescents who are changing schools around age 10 in Germany [26] can face social challenges and increased academic pressure, among others. This transitional life event is likely accompanied by increased stress and has been linked to an increased risk of experiencing mental health challenges [27, 28].

A superior fit in relative fit statistics of the three global scales (INT, EXT, and CON), compared to the disorder-specific seven-factor solution, supports a transdiagnostic approach to early screening, particularly in young PTB adolescents. The meta-analytic finding of overlapping peak onset ages of most mental disorders during adolescence corroborates this idea, as the overlap hinders establishing exact diagnostic categories during this developmental phase [18, 20].

This does not imply that the seven-factor solution, and thus the primary subscales, should be neglected, as fit indices showed similar moderate fit to our data. A closer look at the primary subscales revealed that most subscales’ internal consistency was comparable to the community norm sample from the instrument’s manual [31]. Nevertheless, we observed considerably lower internal consistency on the primary subscales for ASDs and disorders of social behavior across 10- to 14-year-olds (SB; all values > .54). While this can be considered an epiphenomenon of the inferior relative model fit of the disorder-specific solution, it is noteworthy that the affected scales aim to examine social traits and behavior. Responses may have differed due to our altered administration of reading the questions out loud and collecting verbal responses. This administration may have altered social responses due to a social-desirability bias or challenges experienced when responding to a person. As well, in a few cases of individuals with considerable cognitive challenges (*N* = 10), we had to rely on external assessments. It is also conceivable that the observed discrepancy originates from the GNN cohort’s characteristics, as the majority of our sample was born extremely (GA < 28, *N* = 117) or very (GA = 28–32, *N* = 92) preterm, with only a few participants born moderately (GA = 32–34, *N* = 15) or late (GA > 34, *N* = 1) preterm. Later-born individuals had low birth weight in particular. This disadvantage at birth has been associated with challenges in social skills and competence in very preterm-born individuals, as endorsed by parent, teacher, and self-ratings [53, 54].

Contrarily, the primary subscale for OCD shows substantially increased internal consistency in our PTB sample. In PTB populations, evidence for higher odds of developing OCD and a dose–response relationship exists but is moderate [55]. It is conceivable that the association of preterm birth with relatively narrower neurodevelopmental pathways (e.g., executive control, cognitive rigidity, uncertainty tolerance, and sensory sensitivity) and increased symptoms of ASD results in tighter clustering of OCD symptoms in this population [15, 56, 57]. Particularly, symptoms of ASD and OCD have been linked [58].

Lastly, when considering the suitability of the DISYPS-III for examining mental health outcomes in PTB populations, the instrument’s modularity, which goes beyond the screening questionnaires evaluated herein, is a benefit. It provides validated questionnaires for assessing all primary subscales in detail. While not examined in the present study, their existence provides a foundation for detailed evaluations of rather disorder-specific symptoms in PTB populations and could support clinical practice.

Applying the DISYPS-III screening instrument to PTB adolescents from Germany revealed significantly elevated mental health challenges compared to the norm sample. We observed this significant increase on all three global subscales and all but the ASD, depression, and OCD primary subscales. This finding is in line with the literature suggesting preterm birth and low birth weight as risk factors for developing mental disorders [4, 6, 59–61] and having increased odds of developing even multiple psychiatric disorders [4, 5, 59].

The observed pattern of mental health challenges across all PTB adolescents did not provide evidence for a full PBP [2, 10]. Instead, only symptoms of ANX and ASD were directly associated with the same factor by the EFA, but not conjointly with ADHD. Conceivable reasons include the possibility that a disorder-specific examination may be secondary to a more transdiagnostic approach at this young age, introducing more variance and resulting in no consistent associations across the group [18].

In fact, symptoms of ADHD were largely elevated and most frequently experienced within our PTB sample. Since symptoms of DVMs were also substantially elevated in the sampled PTB adolescents, it may simply be that these common developmental alterations drive differences in our sample, but with variance that is independent of reported anxiety and ASD symptoms. Interestingly, we also observed that adolescents diagnosed with the inattentive presentation (ADD) of ADHD reported a substantially increased frequency of comorbid symptoms across all primary subscales, an effect documented in the literature [62, 63]. Replicating this effect provides more evidence for the DISYPS-III’s suitability for PTB populations.

Previous research attributes a central role in driving ADHD prevalence to white matter injuries, a frequent comorbidity of preterm birth [64]. Altered cortical organization due to too early extra-uterine exposure, hypoxia-ischemia, and inflammation can all lead to white matter injuries, which are then associated with problems in sensory processing [65]. Furthermore, PTB infants are exposed to intense stimulation in the neonatal intensive care unit (NICU), a hectic and noisy environment. They have to endure constant nursery handling and the repetitive pain of different treatments, likely leading to overstimulation [66]. At the same time, being separated from their parents due to the incubator can lead to tactile understimulation. In these conditions, PTB circumstances might alter their sensory processing to cope with the NICU environment, which can later become maladaptive and might possibly manifest in symptoms of ADHD [65, 67]. In contrast, individuals without high levels of hyperactivity might be overlooked more easily. Their pattern of symptoms could be rooted in the conceptualization of hyperactivity as an externalizing regulation strategy (e.g., [68]), which leaves adolescents with an inattentive presentation (ADD) more likely to turn to internalizing coping mechanisms and may make them susceptible to symptoms of internalizing disorders.

Differences between internalizing and externalizing strategies might be especially relevant in the present sample, since we found a sex difference specific to internalizing problems. Only these problems were considerably elevated in females, which may underline that female PTB adolescents are more likely to face internalizing mental health problems. Recent evidence from the US has pointed to gender differences in internalizing symptoms also in other populations [5, 69]. This raises awareness of internalizing problems, particularly in girls, across populations and suggests that the observed sex difference can be independent of the general level of mental health challenges, which is elevated in PTB adolescents. Reasons for this difference might be found in gender-specific (social) media use or increased internally focused “coping” strategies [70, 71].

Other factors, including genetic, familial, and the COVID-19 pandemic, may have also played a role in shaping our results, as the participants of the present study were affected by the pandemic at ages 5–9. Though symptoms of DEP and ANX, which have mainly been observed as the pandemic’s consequences [72], were not specifically elevated in our sample. This speaks against the COVID-19 pandemic driving the reported effects in the examined adolescents.

The present results of similar levels of mental health challenges reported by 10-year-old PTB individuals support the call for even earlier screening action [22]. Since emerging symptoms can already signal the onset of latent prodromal phases of psychiatric disorders, screening and primary prevention build the basis for improving psychological long-term outcomes of this at-risk group. Implementing a transdiagnostic screening of long-term mental health outcomes after PTB following selective prevention approaches [73] appears sensible for improving early detection and outcomes during adolescence – a tremendously challenging time for mental health [18, 25]. Moreover, evidence suggests that elevated childhood symptoms are more predictive of adult mental disorders than formal clinical diagnoses alone [74, 75], which underscores the utility of applying screening and intervention already during the primary school years, targeting sub-clinical symptoms before they manifest as diagnosable conditions [76]. While our findings indicate that the DISYPS-III factor structure requires careful interpretation in PTB populations, it remains a suitable tool for identifying the broad architecture of mental health symptoms. Such early identification is particularly critical in PTB populations, who frequently present with specific social and emotional difficulties [2]. Being validated with adolescents and adults also allows for the use of the DISYPS-III during longer follow-up intervals in PTB populations, which have recently been recommended [9].

The results of the present study should be interpreted considering several limitations. First, the study design was merely observational. Since the comparison to the DISYPS-III community norm sample could have included unassessed confounders, such as sampling before the pandemic, all associations should be interpreted without drawing conclusions about causality. Specifically, recent claims about a “youth mental health crisis” suggest that mental health challenges are at an all-time high among adolescents globally [22]. Without assessing a full-term control group, elevated symptom burden in PTB adolescents during 2025–2026, who were compared to a norm sample from 2016, does not conclusively answer whether PTB led to overall increased mental health challenges in our sample. Another aspect of the norm group is represented by some analyses relying on simulated single-participant values and distributions using the given characteristics (N, M, and SD) provided by the manual. Although these simulations may have introduced unquantified biases, our final interpretation tries to account for this uncertainty by being grounded in both parametric and non-parametric inference. Second, since all participants were born and raised in Germany (i.e., a high-income country), generalizability to other areas of the globe may be limited. The most preterm births occur in southern Asia and sub-Saharan Africa, with considerably reduced survival rates among extremely PTB infants (90% vs. 10% [77]). Third, possible covariates regarding the reasons for preterm birth (maternal infections, stress, nutritional status [78]) and the children’s upbringing (e.g., socio-economic status, received support and therapies) were not included in the present analyses and most likely varied between families. To minimize the effects of these limitations, we included a wide range of PTB adolescents across Germany. The multi-centre design allowed for a sample representing various clinics and NICU environments, as well as school systems, region-specific cultures, and socio-economic circumstances. The present sample also includes adolescents with different disabilities, such as cerebral palsy, cognitive impairment, and trisomy 21 (*N* = 10), who are usually excluded or underrepresented in broad scientific samples. Fourthly, to represent the latter adolescents, it became necessary to draw back on external assessments, which constituted a deviation from the standardized self-rating procedure. This may have introduced a bias due to the shift in perspective. Nevertheless, most mental health outcomes were self-rated by the adolescents (96% of participants). This is an improvement over many previous studies, which have exclusively relied on the external assessment of caregivers to operationalize mental health status. Self-rated data carry the potential to draw a more complete picture, including the adolescents’ experiences at school, friends’ houses or sports clubs, and challenges not shared with their parents. Lastly, clear deviations of self-ratings from externally observable behavior led to clinician-adjusted ratings (<1%). Hence, while most ratings comprise solely self-reported data, a minority also follows a combined assessment allowed by the DISYPS-III manual and outlined in the Supplementary Materials. Despite differences in the DISYPS-III implementation, our sample size allows for construct validation of the screening instrument with reasonable statistical power.

In sum, the present study confirms the use of the DISYPS-III screening instrument for assessing mental health outcomes in young adolescents aged 10–14 who were born preterm or with low birth weight (PTB). Specifically, the present study provides qualified support for extending existing validations of this screening instrument to 10-year-olds and under administration conditions that were adapted to the specific needs of a PTB sample. Importantly, young PTB adolescents reported significantly more mental health challenges than the instrument’s community norm sample, with symptoms of ADHD and DVMs being most prominent. Our findings also replicate increased comorbid symptoms reported by individuals with a diagnosis of the inattentive presentation (ADD) of ADHD, compared to those with an ADHD diagnosis including hyperactivity. Though the PTB group’s mental health challenges could not be summarized as a full PBP. Particularly, similar levels of mental health challenges reported by 10-year-old PTB adolescents, compared to their older peers, underline the need to screen PTB individuals for symptoms of mental health disorders as early as age 10. The DISYPS-III, including our adapted administration, represents an applicable and feasible instrument for routine screening and primary prevention. Its design as a broad screening tool allows it to cover a wide range of mental health challenges, including subclinical levels that might otherwise remain unrecognized. Future studies should focus on providing a detailed understanding of the mechanisms linking mental health outcomes to PTB-specific risk factors, including genetics, in utero aberrancies, birth circumstances and complications, birth order, and brain age during adolescence.

## Supporting information

10.1192/j.eurpsy.2026.12240.sm001 Greif et al. supplementary material Greif et al. supplementary material

## Data Availability

All data generated and analysis scripts will be available from the project’s Open Science Framework repository (https://osf.io/rjtep/).

## Supplementary material

The supplementary material for this article can be found at http://doi.org/10.1192/j.eurpsy.2026.12240.

## Long descriptions

### Table 1. Long description

The table presents demographic data for a total N of 225. Columns are divided into Total, Sex (Female N equals 96, Male N equals 129), and Age (Age 10 N equals 46, Age greater than or equal to 11 N equals 179).

Key data points include:

* Age, M (S D): Total 12.03 (1.24); Female 12.11 (1.33); Male 11.97 (1.17); Age 10 10.48 (.32); Age 11 plus 12.42 (1.07).

* G A, M (S D): Total 28.03 (2.49) weeks.

* Birth weight, M (S D): Total 999.62 (296.30) grams.

* Birth weight percentile, M (S D): Total 41.12 (28.42).

* Multiple birth status: Single births account for 54.67 percent (123); Twins 34.22 percent (77); Triplets 6.67 percent (15).

* Clinical Diagnoses: Dyslexia 10.2 percent (23); A D H D 7.1 percent (16); Inattentive presentation diagnosis (A D D) 8.9 percent (20).

Navigate back to Table 1..

### Figure 1. Long description

The diagram consists of two panels, A and B.

Panel A shows a three-factor solution. At the bottom are three ovals representing latent factors, each with a double-headed curved arrow underneath. From left to right:

* E X T points upward to a rectangle labeled 1-9.

* I N T points upward to a rectangle labeled 10-30.

* C O N points upward to a rectangle labeled 36-40 and also has a diagonal arrow pointing to a sub-section of the I N T rectangle labeled 13-15.

Panel B shows a seven-factor solution. At the bottom are seven ovals representing latent factors, each with a double-headed curved arrow underneath. From left to right:

* A D H D points upward to a rectangle labeled 1-3.

* S B points upward to a rectangle labeled 4-9.

* A N X points upward to a rectangle labeled 10-21.

* D E P points upward to a rectangle labeled 22-30.

* D V M points upward to a rectangle labeled 31-35.

* A S D points upward to a rectangle labeled 36-39.

* O C D points upward to a rectangle labeled 42-44.

Navigate back to Figure 1..

### Table 2. Long description

The table contains 8 columns: C F A, Sample, C F I, T L I, R M S E A, S R M R, A I C, and B I C.

Row 1: 3 factors, Sample 10 to 14, C F I .746, T L I .727, R M S E A .060 (bold), S R M R .072 (bold), A I C 14515, B I C 14884.

Row 2: 7 factors, Sample 10 to 14, C F I .766, T L I .747, R M S E A .053 (bold), S R M R .070 (bold), A I C 17904, B I C 18396.

Row 3: 3 factors, Sample 11 plus, C F I .717, T L I .698, R M S E A .064, S R M R .078 (bold), A I C and B I C are blank.

Row 4: 7 factors, Sample 11 plus, C F I .720, T L I .697, R M S E A .060 (bold), S R M R .077 (bold), A I C and B I C are blank.

Note: Bold values indicate acceptable fit. N equals 225 for the 10 to 14 age group and N equals 179 for the 11 plus age group.

Navigate back to Table 2..

### Table 3. Long description

The table contains 10 columns: Scale, M sub all (norm sample), S D sub all (norm sample), 95% C I lower, 95% C I upper, Internal consistency (alpha sub all), alpha sub 11+ (norm sample), Delta alpha (alpha sub 11+ vs. alpha sub all), and Delta M (M sub all vs. M sub norm).

* Total: M sub all .47 (.33), S D sub all .29 (.26), alpha sub all .90, Delta M .14.

* E X T (Externalizing): M sub all .57 (.36), S D sub all .41 (.34), alpha sub all .72, Delta M .21.

* I N T (Internalizing): M sub all .51 (.38), S D sub all .36 (.34), alpha sub all .85, Delta M .13.

* C O N (Attachment): M sub all .34 (.23), S D sub all .34 (.34), alpha sub all .63, Delta M .11.

* A D H D: M sub all 1.01 (.59), S D sub all .77 (.56), alpha sub all .70, Delta M .42.

* A N X (Anxiety): M sub all .56 (.40), S D sub all .39 (.35), alpha sub all .77, Delta M .16.

* A S D (Autism): M sub all .40 (.23), S D sub all .47 (.40), alpha sub all .54, Delta M .17.

* D E P (Depression): M sub all .43 (.37), S D sub all .42 (.41), alpha sub all .76, Delta M .06.

* D V M (Developmental): M sub all .51 (.20), S D sub all .47 (.30), alpha sub all .51, Delta M .31.

* O C D: M sub all .40 (.26), S D sub all .54 (.40), alpha sub all .61, Delta M .14.

* S B (Social Behavior): M sub all .35 (.24), S D sub all .33 (.30), alpha sub all .59, Delta M .11.

Values in brackets represent the official norm sample (N = 950).

Navigate back to Table 3..

### Table 4. Long description

The table is organized into 12 columns. The first column lists the age groups and statistical metrics, followed by a Total column, three Global subscales (E X T, I N T, C O N), and seven Primary subscales (A D H D, A N X, A S D, D E P, D V M, O C D, S B).

* Row 1: Age 10 M (S D). Total .44 (.30). Global: E X T .54 (.39), I N T .48 (.35), C O N .32 (.35). Primary: A D H D 1.00 (.77), A N X .57 (.40), A S D .39 (.46), D E P .35 (.36), D V M .45 (.54), O C D .38 (.40), S B .32 (.31).

* Row 2: Age 11 plus M (S D). Total .48 (.29). Global: E X T .57 (.41), I N T .52 (.37), C O N .35 (.34). Primary: A D H D 1.01 (.77), A N X .56 (.39), A S D .41 (.47), D E P .46 (.44), D V M .52 (.45), O C D .41 (.57), S B .35 (.33).

* Row 3: Delta M (Difference in means). Total .04. Global: E X T .03, I N T .04, C O N .03. Primary: A D H D .01, A N X minus .01, A S D .02, D E P .09, D V M .07, O C D .03, S B .03.

* Row 4: p-value. Total .365. Global: E X T .593, I N T .529, C O N .638. Primary: A D H D .942, A N X .868, A S D .775, D E P .09, D V M .404, O C D .664, S B .472.

* Row 5: Bayes Factor. Total .262. Global: E X T .201, I N T .211, C O N .198. Primary: A D H D .178, A N X .180, A S D .184, D E P .531, D V M .264, O C D .200, S B .222.

Navigate back to Table 4..

### Figure 2. Long description

Panel A, titled D I S Y P S Total Score Quotients split by age, features two horizontal raincloud plots. The y-axis labels the age groups as 10 and greater than or equal to 11. The x-axis represents D I S Y P S Total Quotients from 0.0 to 1.5. The top plot for age 11 plus is yellow, showing a dense cluster of data points and a boxplot centered around 0.5. The bottom plot for age 10 is purple, showing a similar distribution but with a slightly lower median.

Panel B, titled Bootstrapped Means (age less than 11), is a purple histogram showing the frequency of 1000 bootstrapped means on the y-axis (0 to 50) against D I S Y P S Total Quotients on the x-axis (0.30 to 0.55). The distribution is bell-shaped, centered near 0.44. Four vertical dashed lines intersect the data. From left to right: a gray line at 0.36, a black line at 0.44 representing the grand mean for 10-year-olds, an orange line at approximately 0.48 representing the grand mean for the 11 plus group, and a final gray line at 0.52. The gray lines represent the 95 percent confidence interval for the younger group.

Navigate back to Figure 2..

### Table 5. Long description

The table presents statistical differences across eleven scales: Total, E X T, I N T, C O N, A D H D, A N X, A S D, D E P, D V M, S B, and O C D.

* P T B Sample: Mean values range from .34 for C O N to 1.01 for A D H D. Sample size N is 225 for most scales, 224 for D V M, and 214 for O C D.

* Norm Sample: Mean values range from .20 for D V M to .59 for A D H D. Sample size N is 950 for all scales.

* Welch t-test: Shows significant differences (p < .0001) for all scales except D E P (p = .054). The highest T-value is 9.43 for D V M.

* Welch t-test Delta 95% C I: Confidence intervals are provided for all scales, such as [.31, .53] for A D H D.

* Wilcoxon Rank Sum Test: W-values and p-values are listed, with significant differences (p < .0001) for most scales. A S D (p = .069) is not significant.

* Wilcoxon Delta Estimated 95% C I: Estimated differences range from -.33 for A D H D to .003 for O C D.

* Permutation M (Fisher-Pitman): Z-values and p-values show significant differences across all scales, with the highest Z-value of 11.63 for D V M.

* Permutation Med. (Brown-Mood): Z-values and p-values show significant differences for most scales, though O C D (p = .5004) is not significant.

Navigate back to Table 5..

### Figure 3. Long description

The figure consists of four rows of paired plots. The left column (A, C, E, G) features raincloud plots with orange P T B data on top and blue Norm Control data on bottom. Each raincloud includes a density distribution, a boxplot showing median and interquartile range, and individual data points. The right column (B, D, F, H) features line graphs showing the difference in quantiles between P T B and norm samples.

* Row 1 (A and B): Total Quotients. Panel A shows P T B has a wider distribution and higher median than Norm. Panel B shows a positive linear trend in quantile differences from 0.1 to 0.25 as quantiles increase from 0.2 to 0.9.

* Row 2 (C and D): External Quotients (E X T). Panel C shows P T B distribution is shifted right with more extreme outliers. Panel D shows a steep increase in quantile difference, plateauing around 0.3 at the higher deciles.

* Row 3 (E and F): Internal Quotients (I N T). Panel E shows similar medians but a longer right tail for P T B. Panel F shows a stable positive difference around 0.1 to 0.2 across all quantiles.

* Row 4 (G and H): Contact Quotients (C O N). Panel G shows P T B has a higher median and broader spread. Panel H shows the difference starts near zero for the lowest quantiles and rises to a plateau of approximately 0.2 beyond the 0.4 quantile mark.

In all shift functions, the vertical error bars representing 95 percent confidence intervals remain above the zero line for most deciles, indicating statistically significant higher scores for the P T B group.

Navigate back to Figure 3..

### Table 6. Long description

The table is organized into 12 columns. The first column lists the metrics: Male M (S D), Female M (S D), Delta M, p-value, and Bayes Factor. The subsequent columns are Total, Global subscales (E X T, I N T, C O N), and Primary subscales (A D H D, A N X, A S D, D E P, D V M, O C D, S B).

* Male M (S D): Total .45 (.26), E X T .59 (.40), I N T .44 (.30), C O N .35 (.32), A D H D 1.07 (.75), A N X .50 (.35), A S D .43 (.49), D E P .35 (.31), D V M .56 (.51), O C D .34 (.52), S B .35 (.32).

* Female M (S D): Total .50 (.33), E X T .53 (.41), I N T .61 (.42), C O N .34 (.36), A D H D .92 (.79), A N X .65 (.43), A S D .37 (.44), D E P .54 (.52), D V M .45 (.41), O C D .48 (.56), S B .34 (.33).

* Delta M: Total .05, E X T minus .06, I N T .17, C O N minus .01, A D H D minus .15, A N X .15, A S D minus .06, D E P .19, D V M minus .11, O C D .14, S B minus .01.

* p-value: Total .187, E X T .283, I N T .001, C O N .814, A D H D .145, A N X .004, A S D .384, D E P .002, D V M .082, O C D .065, S B .793.

* Bayes Factor: Total .353, E X T .255, I N T 36.37, C O N .151, A D H D .407, A N X 9.56, A S D .208, D E P 31.09, D V M .564, O C D .787, S B .152.

Navigate back to Table 6..

### Figure 4. Long description

A multi-panel figure with six sections labeled A through F.

Panel A and B: Raincloud plots showing D I S Y P S-I I I total and internalizing quotients. The y-axis splits data by males and females. Both show orange density distributions and boxplots with medians around 0.5. Data points are scattered below the density curves.

Panel C: A grouped bar chart comparing Mean Quotients across seven subscales: A D H D, A N X, A S D, D E P, D V M, S B, and O C D. Blue bars represent the Norm Sample and orange bars represent the P T B Sample. The P T B Sample shows significantly higher mean quotients in all categories except D E P, which is marked n s for non-significant. Asterisks indicate significance.

Panel D: A radar plot with a central anchor of 0 and concentric rings for 1 and 2. It maps the same seven subscales. The orange P T B sample line (N = 225) forms a larger polygon than the blue Norm Sample line (N = 950), particularly extending further toward the A D H D and A N X vertices.

Panel E: A raincloud plot specifically for A D H D Quotients. The top orange section represents the P T B Sample with a median near 0.7. The bottom blue section represents the Norm Sample with a lower median near 0.4.

Panel F: A radar plot comparing P T B adolescents by diagnosis. It features three layers: a dark green line for no diagnosis (N = 189), a medium green line for A D H D (N = 16), and a light green line for A D D (N = 20). The A D H D and A D D groups show much larger polygons, indicating higher mean quotients across all subscales compared to the no diagnosis group.

Navigate back to Figure 4..

### Figure 5. Long description

Panel A is a histogram for the dyslexia diagnosis group (N equals 23). The x-axis is D I S Y P S total ranging from 0.00 to 1.25, and the y-axis is Frequency from 0 to 6. The distribution is multimodal with a primary peak at 0.6.

Panel B is a histogram for the diagnosed or suspected dyslexia group (N equals 62). The x-axis ranges from 0.0 to 1.5. The distribution is right-skewed with a prominent peak around 0.3 to 0.4.

Panel C is a radar plot with six axes: A D H D, O C D, A S D, D V M, D E P, A N X, and S B. Concentric rings represent quotients 0, 1, and 2. A red line (dyslexia N equals 23) shows higher mean quotients than the black line (no dyslexia N equals 193), particularly on the A D H D axis.

Panel D is a radar plot with the same six axes. It compares three groups: no dyslexia (black line, N equals 193), dyslexia only (red line, N equals 14), and comorbid A D (H) D (green line, N equals 9). The comorbid group shows the highest quotients across most scales, especially A D H D and S B, while the dyslexia-only group shows intermediate elevations compared to the no-dyslexia control.

Navigate back to Figure 5..
