# Supplementary material for: Mental health outcomes in young adolescents born preterm reveal the need for earlier screening
Source: Eur Psychiatry. 2026 Jul 17;69(1):e78. doi: 10.1192/j.eurpsy.2026.12240 (PMC13458301; doi:10.1192/j.eurpsy.2026.12240)
Supplement: Supplementary file 1 [file S0924933826122408sup001.docx]

Supplementary Material for

Mental health outcomes in young adolescents born preterm reveal need for earlier screening

Emilia Greif^1,2*^, Frederike Schröpfer^1,2*^, Jekaterina Dudko^1^, Leonie Göbel^3^, Rabea Kühl^1^, Katharina Röse^3^, Michael Lipp^4^, Jonas Obleser^2,5^, Wolfgang Göpel^2,6^, Stefan Borgwardt^1,2^, Léon Franzen^1,2#^

^1^ Department of Psychiatry and Psychotherapy, University of Luebeck, Luebeck, Germany

^2^ Center of Brain, Behavior and Metabolism (CBBM), University of Luebeck, Luebeck, Germany

^3^ Institute of Health Sciences – Department of Occupational Therapy, University of Luebeck, Luebeck, Germany

^4^ Diakonie Nord Nord Ost, Fachklinik für Junges Leben, Luebeck, Germany

^5^ Department of Psychology, University of Luebeck, Luebeck, Germany

^6^ Department of Paediatrics, University of Luebeck, Luebeck, Germany

* These authors contributed equally to this work

**# Corresponding author: Léon Franzen, Department of Psychiatry and Psychotherapy, University of Luebeck, Luebeck, Germany; leon.franzen@mail.com**

**S1. Decision criteria for adjusting self-ratings and ratings provided by external, close caregivers on the DISYPS-III**

The decision-making criteria that we used for making our adjustments were guided by clinical assessments on standard disorder-specific instruments, such as the ADOS-2, and discrepancies between self-ratings and accompanying verbal reports or observed behaviour during the 45-minute test session and questionnaire interaction. The test session also included a pen and paper test requiring the ability to focus on visuo-motor tasks and a standardised reading task while getting their eyes tracked (Frostig Developmental Test of Visual Perception – Adolescents and Adults [FEW-JE] and International Reading Speed Texts [IReST]). These tests were carried out right before administering the DISYPS questionnaire. Other observable OCD patterns or general communication features including gaze, facial expressions, tone of voice, and body language were factored into adjustments to the respective items as well.

More specific decision criteria used for clinician’s adjustments were:

- The item on **attention** (S01) was adjusted if an adolescent required to be given the same instructions more than twice or needed external guidance to refocus on a task repeatedly, accordingly.
- The item on **impulsivity** (S02) was adjusted in case of an adolescent starting with a task before clinicians finished the instructions; showed more than one intentionally disruptive or negative incident; engaged in repeated swearing during the session.
- The item on **hyperactivity** (S03) was adjusted in case an adolescent was sitting in the chair but was repeatedly fidgeting; had difficulty sitting and moved about in the chair; or in or out of the chair; or the level of activity disrupted the assessment.
- The item on **eating and body weight** (S30) was adjusted by clinicians if an adolescent’s body weight was visibly above or below the norm-range but did not report having issues with keeping a normal weight.
- The item on **speech difficulties** (S31) was adjusted if a speech impediment was obvious; non-echoed language was used that consisted mostly of simple phrases or limited variation in pitch and tone; intonation was flat or exaggerated; volume was very quiet or loud; speech was somewhat slow, fast, or jerky.
- The item on **learning disorders and reading skills** (S34) was adjusted if children could not finish reading all 3 standardised texts due to exhaustion or took substantially longer than expected based on their school type and grade.
- The item on **addictive** (gaming) **behaviour** (S49) was adjusted if respondents answered that they encountered real and frequent issues with stopping their sessions, even upon repeated reminders from their parents.

**S2. Parallel analysis and scree plot to extract number of factors for exploratory factor analyses.** Blue triangles indicate factors sorted by their eigenvalue. According to the elbow criterion, three factors should be retained. Red dashed lines indicate resampled / simulated data for the parallel analysis. According to the criterion of each factor explaining more variance than factors in the resampled / simulated data, six factors should be retained.

**S3. Overview of sample sizes used in analyses and corresponding exclusion criteria.**

| Analysis | Initial N | Exclusions / Missing Data | Final N | Rationale |
| --- | --- | --- | --- | --- |
| Total Sample | 225 | 0 | 225 | Initial dataset |
| CFA (Full age span) | 225 | 0 | 225 | Imputation with full information maximum likelihood |
| CFA (Age 11+) | 179* | 0 | 179 | Imputation with full information maximum likelihood |
| T-tests (DVM) | 225 | 1 | 224 | ID 54 (missing DVM quotient) |
| T-tests (OCD) | 225 | 11 | 214 | Missing DISYPS items S41, S42 |
| Radar Plot (Dyslexia) | 225 | 9 | 216 | Missing information on dyslexia |

**S4. Standardised factor loadings resulting from confirmatory factor analyses specified at the 7 primary subscales.** Full information maximum likelihood was used to impute 14 missing values. SB = Social Behaviour, ANX = Anxiety, DEP = Depression, DVM = Developmental Disorders, ASD = Autism Spectrum Disorder.

| Item | description | ADHD | SB | ANX | DEP | DVM | ASD | OCD |
| --- | --- | --- | --- | --- | --- | --- | --- | --- |
| S01 | Inattention | .67 |  |  |  |  |  |  |
| S02 | Impulsivity | .70 |  |  |  |  |  |  |
| S03 | Hyperactivity | .68 |  |  |  |  |  |  |
| S04 | Irritability |  | .54 |  |  |  |  |  |
| S05 | Arguing |  | .75 |  |  |  |  |  |
| S06 | Threatening |  | .39 |  |  |  |  |  |
| S07 | Lying, stealing |  | .46 |  |  |  |  |  |
| S08 | Wrecking |  | .35 |  |  |  |  |  |
| S09 | Skipping school |  | .24 |  |  |  |  |  |
| S10 | Separation anxiety |  |  | .31 |  |  |  |  |
| S11 | General anxiety |  |  | .59 |  |  |  |  |
| S12 | Fear of failure |  |  | .62 |  |  |  |  |
| S13 | Fear of peers |  |  | .26 |  |  |  |  |
| S14 | Fear of adults |  |  | .35 |  |  |  |  |
| S15 | Mutism |  |  | .34 |  |  |  |  |
| S16 | Agoraphobia |  |  | .55 |  |  |  |  |
| S17 | Panic attacks |  |  | .49 |  |  |  |  |
| S18 | Specific phobias |  |  | .47 |  |  |  |  |
| S19 | PTSD |  |  | .50 |  |  |  |  |
| S20 | Hypochondria |  |  | .35 |  |  |  |  |
| S21 | Sleep disorder |  |  | .52 |  |  |  |  |
| S22 | Sadness |  |  |  | .73 |  |  |  |
| S23 | Grumpiness |  |  |  | .63 |  |  |  |
| S24 | Anhedonia |  |  |  | .45 |  |  |  |
| S25 | Fatigue |  |  |  | .49 |  |  |  |
| S26 | Feeling inferior |  |  |  | .63 |  |  |  |
| S27 | Withdrawal |  |  |  | .51 |  |  |  |
| S29 | Psychosomatic pain |  |  |  | .38 |  |  |  |
| S30 | Eating disorders |  |  |  | .49 |  |  |  |
| S31 | Language development |  |  |  |  | .51 |  |  |
| S32 | Fine motoric development |  |  |  |  | .45 |  |  |
| S33 | Motoric development |  |  |  |  | .42 |  |  |
| S34 | Learning difficulties |  |  |  |  | .48 |  |  |
| S35 | Enuresis |  |  |  |  | .42 |  |  |
| S36 | Verbal interaction |  |  |  |  |  | .71 |  |
| S37 | Non-verbal interaction |  |  |  |  |  | .52 |  |
| S38 | Lack of friends |  |  |  |  |  | .59 |  |
| S39 | Repetitive behaviour |  |  |  |  |  | .27 |  |
| S42 | Compulsive actions |  |  |  |  |  |  | .32 |
| S43 | Compulsive thoughts |  |  |  |  |  |  | .75 |
| S44 | tics |  |  |  |  |  |  | .53 |

**S5. Covariance matrix of the 7 primary factors in the confirmatory factor analysis.** Variances of each factor were set to 1. SB = Social Behaviour, ANX = Anxiety, DEP = Depression, DVM = Developmental Disorders, ASD = Autism Spectrum Disorder.

|  | ADHD | SB | ANX | DEP | DVM | ASD | OCD |
| --- | --- | --- | --- | --- | --- | --- | --- |
| ADHD | 1 | .67 | .39 | .42 | .66 | .56 | .38 |
| SB |  | 1 | .37 | .57 | .32 | .20 | .34 |
| ANX |  |  | 1 | .84 | .39 | .58 | .75 |
| DEP |  |  |  | 1 | .27 | .52 | .83 |
| DVM |  |  |  |  | 1 | .78 | .29 |
| ASD |  |  |  |  |  | 1 | .42 |
| OCD |  |  |  |  |  |  | 1 |

**S6.** **Standardised factor loadings resulting from confirmatory factor analyses specified at the 3 global Subscales.** N = 14 datasets were excluded due to missing values for item S41 and item S42. The remaining dataset included N = 209 observations. Covariances between factors can be found at the bottom, variances were set to 1. EXT = Externalising problems, INT = Internalising problems, CON = difficulties with contact.

| Item | description | EXT | INT | CON |
| --- | --- | --- | --- | --- |
| S01 | Inattention | .60 |  |  |
| S02 | Impulsivity | .68 |  |  |
| S03 | Hyperactivity | .64 |  |  |
| S04 | Irritability | .45 |  |  |
| S05 | Arguing | .61 |  |  |
| S06 | Threatening | .36 |  |  |
| S07 | Lying, stealing | .40 |  |  |
| S08 | Wrecking | .32 |  |  |
| S09 | Skipping school | .27 |  |  |
| S10 | Separation anxiety |  | .27 |  |
| S11 | General anxiety |  | .55 |  |
| S12 | Fear of failure |  | .60 |  |
| S13 | Fear of peers |  | .26 | -.01 |
| S14 | Fear of adults |  | .29 | .01 |
| S15 | Mutism |  | .35 | -.01 |
| S16 | Agoraphobia |  | .51 |  |
| S17 | Panic attacks |  | .49 |  |
| S18 | Specific phobias |  | .45 |  |
| S19 | PTSD |  | .48 |  |
| S20 | Hypochondria |  | .35 |  |
| S21 | Sleep disorder |  | .50 |  |
| S22 | Sadness |  | .67 |  |
| S23 | Grumpiness |  | .60 |  |
| S24 | Anhedonia |  | .42 |  |
| S25 | Fatigue |  | .51 |  |
| S26 | Feeling inferior |  | .61 |  |
| S27 | Withdrawal |  | .51 |  |
| S29 | Psychosomatic pain |  | .39 |  |
| S30 | Eating disorders |  | .45 |  |
| S36 | Verbal interaction |  |  | .64 |
| S37 | Non-verbal interaction |  |  | .52 |
| S38 | Lack of friends |  |  | .66 |
| S39 | Repetitive behaviour |  |  | .28 |
| S40 | Not being comforted by others |  |  | .57 |
| EXT |  | 1 | .51 | .49 |
| INT |  |  | 1 | .56 |
| CON |  |  |  | 1 |

**S7a. Sensitivity analyses adjusted full sample versus adjusted self-ratings.** Comparison of CFA fit indices based on the full sample N = 225 to a subsample without CP and trisomy 21 individuals N = 215 (Delta compared to original CFA). Bold highlighting indicates that cut-offs for acceptable fit were met.

| CFA | Sample | CFI | TLI | RMSEA | SRMR |
| --- | --- | --- | --- | --- | --- |
| 3 factors | 10-14 | .725 (-.021) | .706 (-.021) | .065 (.005) | **.074 (.002)** |
| 7 factors | 10-14 | .744 (-.022) | .723 (-.024) | **.057 (.004)** | **.071 (.001)** |
| 3 factors | 11+ | .685 (-.032) | .663 (-.035) | .070 (.006) | .082 (.004) |
| 7 factors | 11+ | .687 (-.033) | .661 (-.036) | .066 (.006) | .**079 (.002)** |

**S7b. Sensitivity analyses adjusted versus unadjusted full sample.** Comparison of CFA fit indices based on adjusted ratings to unadjusted ratings (Delta compared to original CFA). Bold highlighting indicates that cut-offs for acceptable fit were met.

| CFA | Sample | CFI | TLI | RMSEA | SRMR |
| --- | --- | --- | --- | --- | --- |
| 3 factors | 10-14 | .762 (.016) | .744 (.017) | **.057 (-.003)** | **.069 (-.003)** |
| 7 factors | 10-14 | .774 (.008) | .755 (.008) | **.052 (-.001)** | **.068 (-.002)** |
| 3 factors | 11+ | .733 (.016) | .713 (.015) | .062 (-.002) | **.076 (-.002)** |
| 7 factors | 11+ | .719 (-.001) | .696 (-.001) | **.060 (/)** | **.077 (/)** |

**S7c. Sensitivity analyses adjusted full sample versus unadjusted self-ratings.** Comparison of CFA fit indices based on adjusted ratings of the full sample N = 225 to unadjusted self-ratings N = 215 (Delta compared to original CFA). N = 1 missing value was imputed using full information maximum likelihood in all CFAs with unadjusted self-ratings. Bold highlighting indicates that cut-offs for acceptable fit were met.

| CFA | Sample | CFI | TLI | RMSEA | SRMR |
| --- | --- | --- | --- | --- | --- |
| 3 factors | 10-14 | .750 (.004) | .731 (.004) | **.060 (/)** | **.070 (-.002)** |
| 7 factors | 10-14 | .745 (-.021) | .724 (-.023) | **.056 (.003)** | **.069 (-.001)** |
| 3 factors | 11+ | .709 (-.008) | .686 (-.012) | .068 (.004) | **.079 (-.001)** |
| 7 factors | 11+ | .688 (-.032) | .663 (-.034) | .066 (.006) | **.080 (-.003)** |

**S8.** **Raincloud Plot of DISYPS Social Behaviour Score Quotients.** Distribution in the PTB sample (orange, N = 225) compared to the norm sample (blue). Data for the norm sample were simulated with the characteristics given in the manual (N = 950, M = .24, SD = .30). Boxplots display median and interquartile range (median_PTB_ = .33, median_norm_ = .31). PTB = Preterm-born.

**S9.** **Raincloud Plot of DISYPS Anxiety Score Quotients.** Distribution in the PTB sample (orange, N = 225) compared to the norm sample (blue). Data for the norm sample were simulated with the characteristics given in the manual (N = 950, M = .40, SD = .35). Boxplots display median and interquartile range (median_PTB_ = .50, median_norm_ = .46). PTB = Preterm-born.

**S10.** **Raincloud Plot of DISYPS Depression Score Quotients.** Distribution in the PTB sample (orange, N = 225) compared to the norm sample (blue). Data for the norm sample were simulated with the characteristics given in the manual (N = 950, M = .37, SD = .41). Boxplots display median and interquartile range (median_PTB_ = .375, median_norm_ = .46). PTB = Preterm-born.

**S11. Raincloud Plot of DISYPS Developmental Disorder Score Quotients.** Distribution in the PTB sample (orange, N = 224) compared to the norm sample (blue). Data for the norm sample were simulated with the characteristics given in the manual (N = 950, M = .20, SD = .30). Boxplots display median and interquartile range (median_PTB_ = .40, median_norm_ = .29). PTB = Preterm-born.

**S12.** **Raincloud Plot of DISYPS Autism Spectrum Disorder Score Quotients.** Distribution in the PTB sample (orange, N = 225) compared to the norm sample (blue). Data for the norm sample were simulated with the characteristics given in the manual (N = 950, M = .20, SD = .30). Boxplots display median and interquartile range (median_PTB_ = .25, median_norm_ = .37). PTB = Preterm-born.

**S13. Raincloud Plot of DISYPS Obsessive Compulsive Disorder Score Quotients.** Distribution in the PTB sample (orange, N = 214) compared to the norm sample (blue). Data for the norm sample were simulated with the characteristics given in the manual (N = 950, M = .20, SD = .30). Boxplots display median and interquartile range (median_PTB_ = .33, median_norm_ = .38). PTB = Preterm-born

**S14.** **EFA results with number of factors specified to 6.** The extraction method used was maximum likelihood, rotation method was oblimin, allowing for correlations between factors. Correlation matrix is displayed at the bottom of the table. Fit indices were Tucker-Lewis Index = .858, root mean squared residual = .05 and root mean squared error of approximation = .036. Factors can be summarised as F1 = transdiagnostic internalising problems, F2 = Autism Spectrum Disorder, F3 = externalising problems, F4 = anxiety, F5 = internalising developmental difficulties, F6 = externalising developmental difficulties h^2^ = communality, SS loadings = eigenvalue.

| Item | description | F1 | F2 | F3 | F4 | F5 | F6 | h^2^ |
| --- | --- | --- | --- | --- | --- | --- | --- | --- |
| S01 | Inattention |  |  |  |  |  |  | .04 |
| S02 | Impulsivity |  |  | .55 |  |  | .30 | .45 |
| S03 | Hyperactivity |  |  | .43 |  |  |  | .36 |
| S04 | Irritability | .33 |  | .38 |  |  |  | .34 |
| S05 | Arguing |  |  | .72 |  |  |  | .53 |
| S06 | Threatening |  |  | .45 |  |  |  | .34 |
| S07 | Lying, stealing |  |  | .46 |  |  |  | .27 |
| S08 | Wrecking |  |  | .31 |  |  |  | .12 |
| S09 | Skipping school |  | .48 |  |  |  |  | .47 |
| S10 | Separation anxiety |  |  |  | .33 |  | .27 | .22 |
| S11 | General anxiety |  |  |  | .67 |  |  | .55 |
| S12 | Fear of failure |  |  |  | .48 |  |  | .43 |
| S13 | Fear of peers |  |  |  | .30 |  |  | .22 |
| S14 | Fear of adults |  |  |  |  |  |  | .17 |
| S15 | Mutism |  |  |  |  |  |  | .23 |
| S16 | Agoraphobia |  | .30 |  | .26 |  |  | .37 |
| S17 | Panic attacks | .50 |  |  |  |  |  | .33 |
| S18 | Specific phobias |  |  |  | .45 |  |  | .28 |
| S19 | PTSD | .35 |  |  |  |  |  | .29 |
| S20 | Hypochondria |  |  |  | .34 |  |  | .24 |
| S21 | Sleep disorder | .41 |  |  |  |  |  | .30 |
| S22 | Sadness | .64 |  |  | .30 |  |  | .70 |
| S23 | Grumpiness | .31 |  | .37 |  |  |  | .51 |
| S24 | Anhedonia | .26 |  |  |  |  |  | .28 |
| S25 | Fatigue |  |  |  |  |  |  | .30 |
| S26 | Feeling inferior | .47 |  |  |  | .33 |  | .53 |
| S27 | Withdrawal |  | .37 |  |  |  |  | .31 |
| S29 | Psychosomatic pain | .35 |  |  |  |  |  | .23 |
| S30 | Eating disorders | .36 |  |  |  |  |  | .33 |
| S31 | Language development |  |  |  |  |  |  | .21 |
| S32 | Fine motoric development |  | .32 |  |  |  | .26 | .23 |
| S33 | Motoric development |  |  |  |  |  |  | .13 |
| S34 | Learning difficulties |  |  |  |  |  |  | .20 |
| S35 | Enuresis |  |  |  |  | .32 |  | .23 |
| S36 | Verbal interaction | .26 | .52 |  |  |  | .26 | .53 |
| S37 | Non-verbal interaction |  | .73 |  |  |  |  | .52 |
| S38 | Lack of friends |  | .55 |  |  |  |  | .34 |
| S39 | Repetitive behaviour |  |  |  |  | .34 |  | .20 |
| S40 | Not being comforted by others |  | .43 |  |  |  |  | .27 |
| S41 | Seeking attention |  |  |  |  | .61 |  | .46 |
| S42 | Compulsive actions | .43 |  |  |  |  |  | .22 |
| S43 | Compulsive thoughts | .67 |  |  |  |  | .35 | .57 |
| S44 | Tics | .47 |  |  |  |  |  | .40 |
| S45 | Emotional lability | .40 |  | .26 |  |  |  | .51 |
| S46 | Self-harming behaviour | .49 |  |  |  |  |  | .36 |
| S47 | Hallucinations |  |  |  |  |  |  | .24 |
| S48 | Alcohol, drug consumption |  |  |  |  |  |  | .11 |
| S49 | gaming |  | .27 | .50 |  |  |  | .40 |
| SS loading | | 4.11 | 3.28 | 2.92 | 2.64 | 1.55 | 1.33 |  |
| Variance explained | | .09 | .07 | .06 | .05 | .03 | .03 |  |
| Cumulative variance explained | | .09 | .15 | .21 | .27 | .30 | .33 |  |
| Correlation Factor 1 | |  | .29 | .22 | .39 | .14 | .08 |  |
| Correlation Factor 2 | |  |  | .27 | .31 | .18 | .05 |  |
| Correlation Factor 3 | |  |  |  | .16 | .13 | .03 |  |
| Correlation Factor 4 | |  |  |  |  | .17 | .03 |  |
| Correlation Factor 5 | |  |  |  |  |  | .01 |  |
